# B7-H4 expression is upregulated by PKCδ activation and contributes to PKCδ-induced cell motility in colorectal cancer

**DOI:** 10.1186/s12935-022-02567-1

**Published:** 2022-04-11

**Authors:** Bin Zhou, Youwei Lu, Zhiming Zhao, Tongguo Shi, Hongya Wu, Weichang Chen, Liang Zhang, Xueguang Zhang

**Affiliations:** 1 Jiangsu Institute of Clinical Immunology, The First Affiliated Hospital of Soochow University, Suzhou, Jiangsu China; 2 Jiangsu Key Laboratory of Clinical Immunology, Soochow University, Suzhou, Jiangsu China; 3 Jiangsu Key Laboratory of Gastrointestinal Tumor Immunology, Soochow University, Suzhou, Jiangsu China; 4 College of Pharmaceutical Sciences, Soochow University, Suzhou, Jiangsu China

**Keywords:** B7-H4, PKCδ, Regulation, Metastasis, Colorectal cancer

## Abstract

**Introduction:**

B7-H4 is overexpressed in colorectal cancer (CRC) and plays an important role in tumor growth and immunosuppression. However, the exact mechanism that regulates B7-H4 expression remains largely unknown. Here, we investigated whether protein kinase C δ (PKCδ) regulates the expression of B7-H4 in CRC.

**Methods:**

By using immunohistochemical (IHC) and immunofluorescence (IF) staining, we analyzed the expression of B7-H4 and phospho-PKCδ (p-PKCδ) in 225 colorectal tumor samples and determined the clinical significance of the expression patterns. In vitro experiments were performed with the CRC cell lines HCT116 and SW620 to detect the effect of PKCδ activation on B7-H4 expression, and xenograft-bearing mice were treated with rottlerin to monitor the expression of B7-H4 and tumor metastasis.

**Results:**

The B7-H4 expression level was significantly correlated with the p-PKCδ level (r = 0.378, *P* < 0.001) in tumor tissues. Coexpression of p-PKCδ and B7-H4 was significantly associated with moderate/poor differentiation (*P* = 0.024), lymph node metastasis (*P* = 0.001) and advanced Dukes’ stage (*P* = 0.002). Western blot analysis showed that Phorbol-12-Myristate-13-Acetate (TPA) increased B7-H4 expression in a concentration-dependent manner and that rottlerin abrogated the TPA-induced increase in B7-H4 expression. The protein levels of B7-H4 and p-STAT3 were significantly reduced by a PKCδ-specific siRNA. Moreover, the STAT3 inhibitor cryptotanshinone significantly decreased the B7-H4 protein level in CRC cells. Knockdown of B7-H4 or PKCδ suppressed cell migration and motility. Rottlerin also inhibited B7-H4 expression and tumor metastasis in vivo.

**Conclusion:**

The B7-H4 expression level is significantly correlated with the p-PKCδ level and tumor metastasis in CRC samples. B7-H4 expression is upregulated by STAT3 activation via PKCδ and plays roles in PKCδ-induced cancer cell motility and metastasis, suggesting that the PKCδ/STAT3/B7-H4 axis may be a potential therapeutic target for CRC.

**Supplementary Information:**

The online version contains supplementary material available at 10.1186/s12935-022-02567-1.

## Introduction

Colorectal cancer (CRC) is the third most common cancer worldwide, and approximately 30,000 new cases occur every year. CRC leads to 13,000 deaths annually and is the fourth leading cause of cancer-related mortality [1, 2]. Although advances in early diagnosis and therapeutic strategies have decreased the mortality of CRC, the survival rate is still poor [3, 4]. The high mortality rate is usually attributed to tumor recurrence and metastasis.

B7-H4 (VTCN1/B7x/B7S1) is a costimulatory molecule in the B7 family and is mainly expressed by antigen-presenting cells (APCs). The expansion of both neutrophil progenitors and T cells can be suppressed by B7-H4, resulting in immune suppression [5–7]. In numerous tumor tissues, B7-H4 is overexpressed and positively correlated with various clinicopathological features [8–13]. B7-H4 expression was found to be significantly higher in CRC tissues than in normal tissues and positively related to infiltration depth, lymph node metastasis and regulatory T cell (Treg) infiltration [14, 15]. Furthermore, soluble B7-H4 in serum has been shown to be a potential biomarker for diseases [16–19]. In addition to its role in immune suppression, B7-H4 also promotes tumor proliferation and metastasis, as revealed by many studies. Li et al. found that B7-H4 facilitates the proliferation and metastasis of CRC cells [20]. Zhang et al. revealed that B7-H4 also promotes lung cancer growth and metastatic progression [21]. Xie et al. showed that B7-H4 promotes tumor invasion and metastasis through activation of ERK1/2 signaling [22]. In summary, B7-H4 contributes to immune evasion, tumor growth and tumor metastasis, but the exact mechanism that regulates B7-H4 expression is not well elucidated.

The protein kinase C (PKC) family, comprising a series of serine/threonine kinases, regulates various cellular physiological processes [23, 24]. The PKCδ level is elevated in colorectal cancer tissue, suggesting a specific role for PKCδ in colon carcinogenesis [25–27]. As a unique novel PKC, PKCδ plays a significant role in diverse cancers and has different cell-specific effects [28–30]. PKCδ is proposed to act mainly as a tumor suppressor due to its antiproliferative and proapoptotic activities. However, it was found that downregulation of PKCδ can induce cytotoxicity and inhibit the growth of cultured stem-like cells originating from human breast, pancreatic and prostate cancers[29, 31]. In colon cancer, PKCδ can inhibit cell growth and proliferation, and it also acts as a proapoptotic regulator [32, 33]. Furthermore, many studies have revealed that PKCδ is involved in colon cancer cell migration and invasion [26, 34–36].

In this study, we demonstrated a positive correlation between B7-H4 and p-PKCδ in clinical CRC samples. Furthermore, the p-PKCδ^+^B7-H4^+^ phenotype was associated with tumor metastasis. To verify this finding in clinical samples, we investigated the effect of PKCδ on B7-H4 expression. Activation of PKCδ by TPA enhanced B7-H4 expression. However, interfering with PKCδ expression by transfection of specific small interfering RNA (siRNA) constructs downregulated B7-H4 in colorectal cancer cell lines. Furthermore, both PKCδ and B7-H4 contributed to CRC cell motility. Knockdown of B7-H4 abrogated PKCδ-induced cell metastasis, confirming that B7-H4 mediates PKCδ-induced cell metastasis and invasion.

## Materials and methods

### Clinical samples

The First Affiliated Hospital of Soochow University (Suzhou, China) approved our experimental protocols. A total of 225 colon cancer tissue specimens and 36 adjacent normal tissue specimens were obtained for immunohistochemical (IHC) analysis. All experimental procedures conformed to the tenets of the Declaration of Helsinki (as revised in 2013). Ethical approval of experiments involving clinical samples was given by the Institutional Review Board of Soochow University (No. 2014865082). In addition, informed consent was also obtained from the patients for the experimental use of their samples.

### Immunohistochemical and immunofluorescence staining

For IHC analysis of p-PKCδ, a rabbit monoclonal antibody specific for human p-PKCδ (phospho-S299, ab133456) purchased from Abcam (Cambridge, MA, USA) was used at a 1:100 dilution. For B7-H4 IHC staining, a mouse monoclonal antibody (clone 3C8, Suzhou, China) specific for human B7-H4 was produced in our laboratory and used at a 1:200 dilution [37]. For IF analysis, Alexa Fluor 488-conjugated goat anti-rabbit IgG (1:100, A32731) and Alexa Fluor 594-conjugated goat anti-mouse IgG (1:200, A11005) were used as the secondary antibodies from Invitrogen (Carlsbad, CA, USA).

Sections (4 µm thick) were sliced from paraffin-embedded samples with a Leica microtome (Wetzlar, Germany). Tissue microarrays were deparaffinized, rehydrated, rinsed, and stained for B7-H4 and p-PKCδ as previously described [38]. IHC staining was performed using the ChemMate™ Envision/HRP technique (Gene Tech Company Limited). The sections were evaluated at low magnification (100 ×) to identify positive staining. The samples were classified into four groups according to the percentage of cells positive for B7-H4 or p-PKCδ signals: 1, positive signals in less than 25% of cells; 2, in 26–50%; 3, in 51–75%; and 4, in more than 75%. The staining intensity was categorized based on the relative intensity as follows: 1, weak staining; 2, intermediate staining; and 3, strong staining. The total score was calculated as the positive percentage score Χ the staining intensity score. Cases with a Quickscore of ≥ 4 were considered positive, and the others were regarded as negative. The stained slides were examined and evaluated by two independent investigators.

For IF analysis, serial sections were incubated with monoclonal anti-p-PKCδ, an anti-B7-H4 and/or isotype IgG antibodies for 1.5 h at room temperature, as previously described [38]. An Alexa Fluor 488-conjugated secondary antibody was used to detect p-PKCδ. An Alexa Fluor 594-conjugated secondary antibody was used to detect B7-H4. IF images were acquired under a Leica DM2500 microscope (Wetzlar, Germany).

### Cell culture and transfection

The human CRC cell lines HCT116, SW620, SW480, RKO and NCM460 were purchased from Shanghai Cell Bank (Chinese Academy of Sciences, Shanghai, China). Cells were cultured in RPMI 1640 medium containing 10% fetal bovine serum (FBS) at 37 °C in a humidified atmosphere of 5% CO_2_. RPMI 1640 medium and FBS were purchased from HyClone (Logan, UT, USA). The PKCδ activator TPA, PKCδ inhibitor rottlerin (Santa Cruz Biotechnology, CA, USA) and STAT3 inhibitor cryptotanshinone (Selleckchem, USA) were stored at − 80 °C.

Human PKCδ-specific siRNAs, a human B7-H4-specific siRNA and the corresponding control siRNAs (con siRNAs) were purchased from GenePharma Co. Ltd. (Shanghai, China). SiRNAs were transfected into HCT116 or SW620 cells using Lipofectamine 2000 reagent (Invitrogen, Carlsbad, CA, USA). RT–qPCR and Western blotting were performed to evaluate the transfection efficiency.

### Total RNA isolation and RT–qPCR

Total RNA was isolated using TRIzol reagent (Invitrogen, Carlsbad, CA, USA), incubated for 20 min at 37 °C with RNase-Free DNase, purified with an RNeasy MinElute Cleaning Kit (74204, Qiagen, Hilden, Germany), and quantified with a spectrophotometer (BioDrop-μLite, UK). Total RNA was reverse transcribed into cDNA using PrimeScript™ RT Master Mix (Takara Bio, Japan). A SYBR PrimeScript RT–qPCR Kit was used (Takara Bio, Japan) to examine individual genes. The thermal cycling procedure used for PCR was as follows: 95 °C for 2 min, followed by 45 cycles of denaturation at 95 °C for 10 s, annealing at 59 °C for 40 s and extension at 72 °C for 45 s. The levels of all genes examined were normalized to the GAPDH mRNA level. The primers for the individual genes used in RT–qPCR are listed in Additional file 7: Table S1.

### Protein extraction and Western blot analysis

Human CRC cells were cultured in 6-well plates and were then lysed with RIPA lysis buffer (Beyotime, Shanghai, China). Protease inhibitor cocktail was added to the RIPA buffer. After determining the protein concentrations of all samples with BCA protein assay kits (Beyotime, Shanghai, China), samples containing equal amounts of total protein were loaded onto a gel and separated by electrophoresis. The protein bands on the gel were transferred to polyvinylidene difluoride (PVDF) membranes (Merck Millipore, Germany). The membranes were incubated with primary antibodies at 4 °C overnight. The primary antibodies used were as follows: mouse anti-human B7-H4 (3C8), rabbit anti-human PKCδ (#9616T, CST, Danvers, Massachusetts, USA), rabbit anti-human/mouse p-PKCδ (ab133456, Abcam, USA), rabbit anti-human/mouse STAT3 (#12640, CST), rabbit anti-human/mouse phospho-STAT3 (p-STAT3, Tyr705, #9145, CST), rabbit anti-human/mouse GAPDH (#5174, CST) and rabbit anti-human/mouse β-actin (#4970, CST). After three washes with PBST, the PVDF membranes were incubated with secondary antibodies at room temperature for 2 h. The secondary antibodies were as follows: HRP-conjugated goat anti-mouse/anti-rabbit IgG (H + L) and rabbit anti-goat IgG (H + L) (Thermo Fisher Scientific, Waltham, MA, USA). The membranes were washed with PBST five times. Then, the membranes were immersed in electrochemiluminescence (ECL) detection reagent (CST). Images were acquired with a Gel DocTM EZ System (Bio–Rad, USA). Image Lab 4.0.1 software (Bio–Rad, USA) was used to analyze band intensities.

### Flow cytometric analysis and IF analysis

To examine intracellular B7-H4 expression, cells were permeabilized using Intracellular Fixation & Permeabilization Buffer (eBioscience, CA, USA). Then, the cells were stained with a PE-conjugated anti-B7-H4 antibody (#358104, Biolegend, CA, USA). PE-conjugated mouse IgG1 isotype control (eBioscience) was used as the control antibody. Flow cytometry was performed on a Beckman flow cytometer. Data were analyzed using FlowJo software (version 7.6, Tree Star Inc.).

For IF analysis of B7-H4 expression in cells, cultured cells were fixed with cold acetone for 10 min, washed in PBS buffer containing 1% FCS, and stained with a PE-conjugated anti-B7-H4 antibody.

### Transwell invasion assay

After 16 h of transfection, cells were harvested by trypsin digestion and resuspended in culture medium containing 10% FBS. A total of 5 × 10^4^ HCT116 or 8 × 10^4^ SW620 cells were seeded in 24-well Transwell chambers containing membranes with a pore size of 8 μm (Falcon, USA). The membranes were precoated with 100 μl of a 1:16 dilution of Matrigel (Corning, New York, USA). After 4–6 h, the culture medium in each well was changed to culture medium containing 2% FBS and a drug or solvent. Then, 700 μl of medium containing 20% FBS was added to the bottom compartment to act as a chemoattractant. After culture for 24 h, the cells that invaded to the underside of the Transwell membrane were fixed with methanol and stained with a 0.1% crystal violet solution. Then, the stained cells were observed under a Nikon TI-SR inverted microscope and imaged using a Nikon DS-Fi2 camera. ImageJ was used to determine the number of cells in three different fields of view.

### Wound healing assay

For evaluation of migration by a wound healing assay, 12-well plates were seeded with cells at a density of 3 × 10^5^ cells/well. After growth and attachment overnight, the surface of the cultured cells was scratched with 10-µl pipette tips. After another 24 h of culture, wound closure was observed and imaged using a Nikon DS-Fi2 camera. The wound closure percentage was calculated using ImageJ software.

### Animal experiments

Ten female BALB/c nude mice aged 4–6 weeks were purchased from Shanghai Laboratory Animal Center (Shanghai, China). After 1 week of adaptation, HCT-116 cells (2 × 10^6^) in PBS (100 μl) were injected intravenously into the nude mice. After 24 h, the mice were randomly allocated to two groups: one group of mice was orally administered 200 μl of solvent, while another was orally administered rottlerin (20 mg/kg) once every two days. The dosage of rottlerin was determined according to our preliminary experimental results and previous reports [39, 40]. All procedures in these experiments were approved by the Animal Protection and Use Committee of Soochow University. Fifty-four days after injection, the mice were sacrificed to assess tumor development, and the lungs were removed and embedded in paraffin for further analyses.

### Bioinformatics analysis

Correlations between the expression of PKCs and that of B7-H4 in COAD patient samples was analyzed. The mRNA expression of PRKCD (encoding PKCδ) and VTCN1 (encoding B7-H4) in COAD tissues and normal tissues from the TCGA colon adenocarcinoma (COAD) dataset was compared. These bioinformatic analyses were performed via the Gene Expression Profiling Interactive Analysis (GEPIA) website [41] (http://gepia.cancer-pku.cn). The correlation between the B7-H4 mRNA level and lymph node metastasis status in the TCGA COAD dataset was performed via the XENA website.

### Statistical analysis

Statistical analyses of the experimental results were performed with GraphPad Prism version 5.0. Associations between p-PKCδ expression, B7-H4 expression and various clinicopathological parameters were evaluated by the χ^2^ test. Correlations were evaluated by the Spearman rank correlation coefficient. Differences between groups were evaluated using two-tailed unpaired Student’s t test. Replicate experiments were analyzed using paired Student’s t test. All significance tests were two-tailed, and *P* < 0.05 was considered significant.

## Results

### B7-H4 and p-PKCδ were upregulated in CRC

**Fig. 1:**
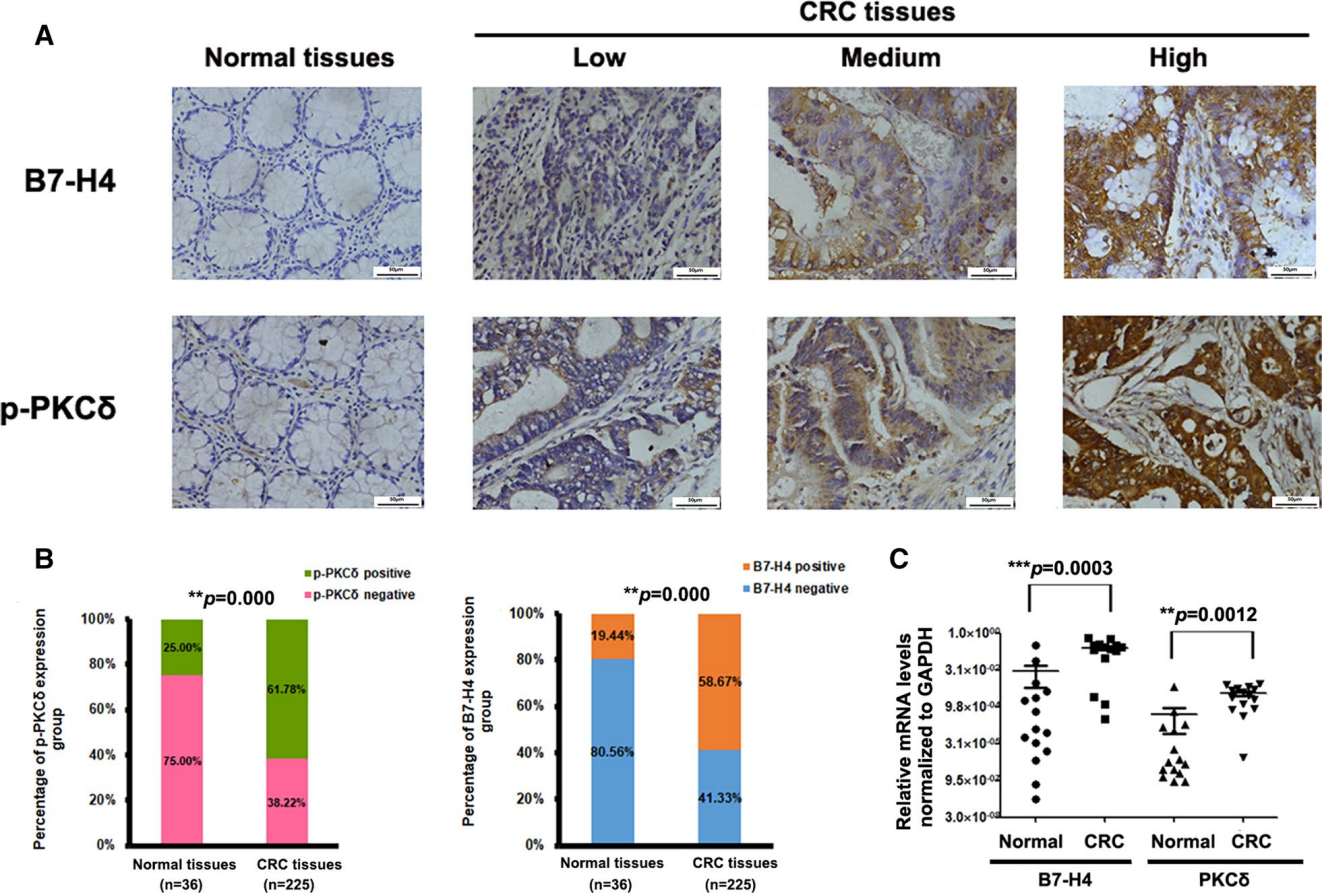
B7-H4 and p-PKCδ levels were increased in clinical CRC tissue specimens. **A** Representative images of negative (400 ×), weak (400 ×), intermediate (400 ×), and strong (400 ×) staining for B7-H4 and p-PKCδ. **B** B7-H4 and p-PKCδ levels were significantly increased in CRC. The levels of B7-H4 and p-PKCδ in CRC tissue samples were compared with those in adjacent normal tissue samples. Statistical analysis was conducted with an unpaired t test (*P* < 0.001). **C** Quantitative PCR analysis showed that the levels of both B7-H4 and PKCδ were significantly increased in tumor tissue samples compared with adjacent normal colon tissue samples

First, we analyzed correlations between the expression of PKCs and that of B7-H4 in CRC patient samples based on the LinkedOmics and GEPIA databases and found that the expression of both PKRCA and PRKCD (PKCδ) was positively associated with B7-H4 expression, but only PKCδ was abnormally upregulated in colorectal cancer tissues (Additional file 1: Figure S1). Then, we evaluated the B7-H4 and p-PKCδ protein levels by IHC staining in 225 clinical colorectal tumor tissue specimens and 36 adjacent normal tissue specimens. Representative images of IHC staining are shown in Fig. 1A. Positive B7-H4 expression was detected in 132 of the CRC tissue specimens (132/225, 58.7%), and B7-H4 was expressed in the membrane, cytoplasm and nucleus of colorectal tumor cells (Fig. 1A). Positive staining for p-PKCδ was detected in 139 of the CRC tissue specimens (139/225, 61.8%), and p-PKCδ was expressed in the membrane and cytoplasm of colorectal tumor cells (Fig. 1A). Comparison of the tumor tissues and adjacent normal tissues revealed that the expression of both B7-H4 and p-PKCδ was significantly increased in the tumor tissue samples (Fig. 1B), consistent with previous reports [14, 25, 27, 40]. Furthermore, quantitative PCR analysis showed that the expression of both B7-H4 and PKCδ was significantly increased in the tumor tissues compared with the adjacent normal tissues (Fig. 1C).

#### The association between B7-H4 and p-PKCδ in CRC

**Fig. 2:**
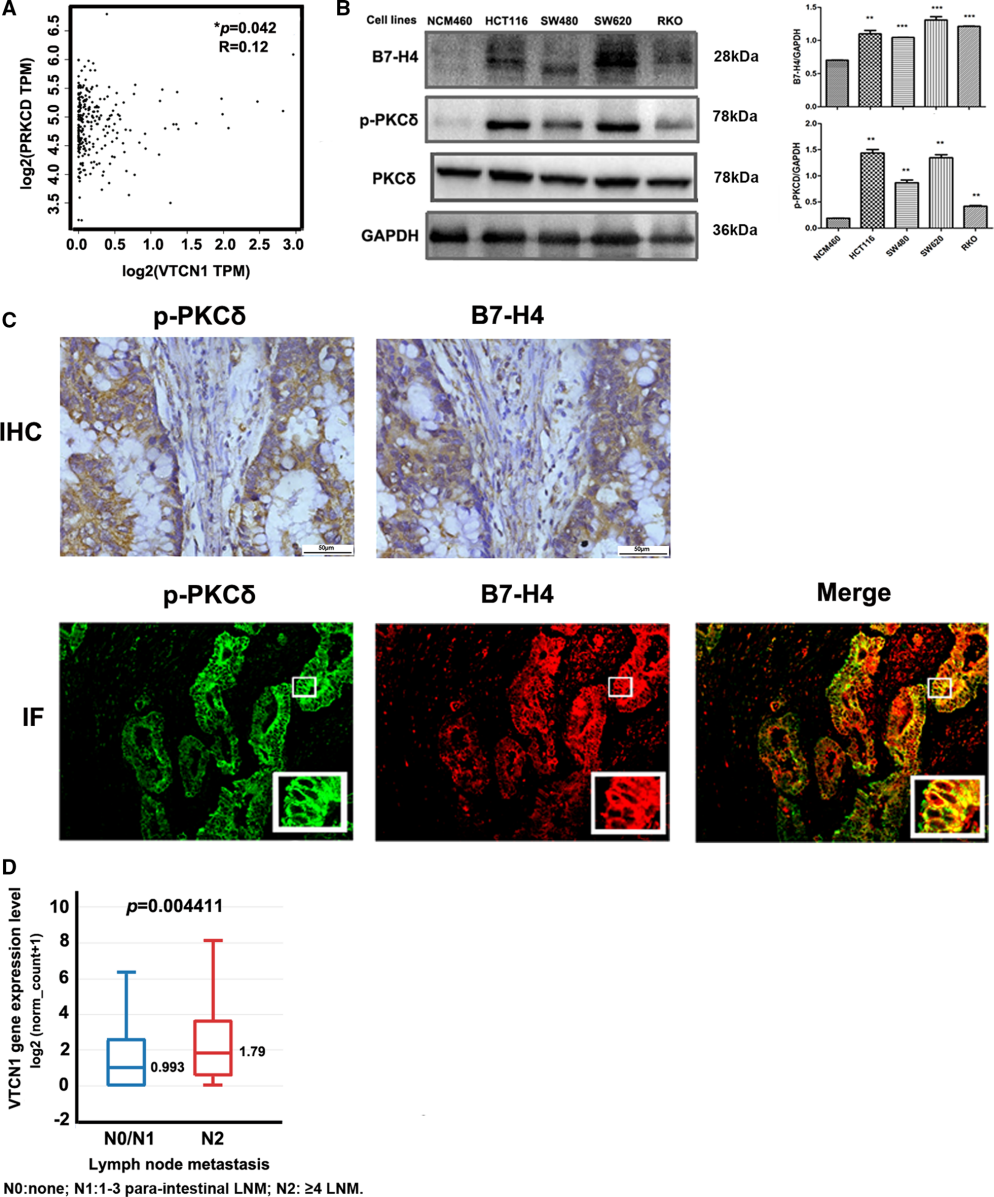
Correlation between B7-H4 and p-PKCδ levels. **A** TCGA dataset analysis was performed on the GEPIA website (http://gepia.cancer-pku.cn/). This analysis showed that PRKCD (PKCδ) mRNA expression was positively correlated with VTCN1 (encoding B7-H4) mRNA expression. **B** The protein levels of B7-H4 and p-PKCδ in the NCM460, SW480, HCT116, SW620 and RKO cell lines were determined by Western blot analysis. The data are expressed as the mean ± SD values; *n* = 3. **C** Serial p-PKCδ^+^ tumor sections were examined for B7-H4 expression, and positive staining (400 ×) of B7-H4 and p-PKCδ in CRC samples is shown. Double IF staining of CRC samples was performed. CRC tissue samples were stained for p-PKCδ (green, 200 ×) and B7-H4 (red, 200 ×). B7-H4^+^/p- PKCδ^+^ cells were identified in CRC tissue specimens (dark yellow; original magnification). **D** TCGA COAD dataset analysis was performed on the XENA website. This analysis showed that the B7-H4 mRNA level was positively correlated with lymph node metastasis

Spearman correlation analysis of the IHC staining data showed that B7-H4 protein expression was significantly correlated with p-PKCδ protein expression (r = 0.378, *P* < 0.001). Analysis with the Pearson chi-square test also showed that B7-H4 protein expression was significantly correlated with p-PKCδ protein expression (*P* < 0.001) (Table 1). Of the 139 p-PKCδ-positive tumor samples, 101 (72.7%, 101/139) showed positive B7-H4 expression, whereas of the 86 p-PKCδ-negative tumor samples, only 31 (36.0%, 31/86) showed positive B7-H4 expression. Analysis of the TCGA colon adenocarcinoma (COAD) dataset showed that the PRKCD mRNA level was also positively correlated with the VTCN1 (encoding B7-H4) mRNA level (Fig. 2A).

**Table 1:** The expression of B7-H4 and p-PKCδ in clinical CRC tissues

p-PKCδ expression	B7-H4 expression (%)		*P* value^a^
	Negative(n = 93)	Positive(n = 132)	
Negative (n = 86)	55 (64.0)	31 (36.0)	0.000^b^
Positive (n = 139)	38 (27.3)	101 (72.7)	

^a^*P* value is obtained by Pearson chi-square, Asymp. Sig., two tailed

^b^*P* < 0.05 was designated as significance

To verify the finding that the B7-H4 protein level is correlated with the p-PKCδ level in clinical samples, we examined B7-H4 and p-PKCδ levels in CRC cell lines. As shown in Fig. 2B and Additional file 2: Figure S2, the B7-H4 protein level was higher in the HCT116 and SW620 cell lines, intermediate in the SW480 and RKO cell lines, and lower in the NCM460 cell line. Consistent with this pattern, the p-PKCδ level was higher in HCT116 and SW620 cells than in the other cells, verifying the correlation between the p-PKCδ and B7-H4 protein levels in CRC cell lines.

Collectively, the results obtained from analysis of clinical CRC samples and CRC cell lines showed that the B7-H4 level was correlated with the p-PKCδ level in CRC.

As we found that the B7-H4 protein level was significantly correlated with the p-PKCδ level, we further investigated whether B7-H4 and p-PKCδ are coexpressed in CRC tissue. Serial sections of p-PKCδ^+^B7-H4^+^ CRC samples were subjected to a serial staining protocol as follows: IHC staining for p-PKCδ, IHC staining for B7-H4, and double IF staining for p-PKCδ and B7-H4. Representative images of stained sections are shown in Fig. 2C. IHC staining showed that the area of positive staining was similar for the two molecules. Merged images of double IF staining showed that the green and red fluorescence signals overlapped. Both the IHC and IF staining results demonstrated that p-PKCδ and B7-H4 were coexpressed in CRC tissue samples.

The results of serial CRC sample staining further indicated that B7-H4 expression in CRC tissues was correlated with PKCδ activation.

### Positivity for B7-H4 and p-PKCδ was associated with tumor metastasis in CRC samples

Next, we examined the associations of B7-H4 and p-PKCδ with clinical parameters. As shown in Table 2, in the 225 CRC samples, positive B7-H4 expression was correlated with moderate/poor differentiation (χ2 = 8.992, *P* = 0.003), lymph node metastasis (χ^2^ = 8.919, *P* = 0.003) and advanced Dukes’ stage (χ^2^ = 5.427, *P* = 0.02) and that positivity for p-PKCδ was correlated with advanced Dukes’ stage (χ^2^ = 4.118, *P* = 0.042). Notably, coexpression of B7-H4 and p-PKCδ was significantly associated with moderate/poor differentiation (χ^2^ = 5.072, *P* = 0.024), lymph node metastasis (χ^2^ = 10.909, *P* = 0.001) and advanced Dukes’ stage (χ^2^ = 10.017, *P* = 0.002). TCGA COAD dataset analysis via the XENA website showed that the B7-H4 mRNA level was also positively correlated with lymph node metastasis (Fig. 2D). These results suggested that B7-H4 and p-PKCδ were associated with tumor metastasis.

**Table 2:** The association of B7-H4 and p-PKCδ with clinical parameters

Variables	B7-H4 (%)			p-PKCδ (%)			p-PKCδ^+^B7-H4^+^ (%)		
	Positive (n = 132)	Negative (n = 93)	*P* value^a^	Positive (n = 139)	Negative (n = 86)	*P* value^a^	Positive (n = 101)	Others (n = 124)	*P* value^a^
Gender									
Male (n = 122)	72 (59.0%)	50 (41.0%)	0.908	78 (63.9%)	44 (36.1%)	0.469	53 (43.4%)	69 (56.6%)	0.635
Female (n = 103)	60 (58.3%)	43 (41.7%)		61 (59.2%)	42 (40.8%)		48 (46.6%)	55 (53.4%)	
Histology									
Well (n = 36)	13 (36.1%)	23 (63.9%)	0.003^b^	22 (61.1%)	14 (38.9%)	0.928	10 (27.8%)	26 (72.2%)	0.024^b^
Moderate/poor (n = 189)	119 (63.0%)	70 (37.0%)		117 (61.9%)	72 (38.1%)		91 (48.1%)	98 (51.9%)	
Depth of tumor									
T1 + T2 (n = 51)	26 (51%)	25 (49%)	0.205	31 (52.6%)	20 (47.4%)	0.868	19 (15.8%)	32 (84.2%)	0.213
T3 + T4 (n = 174)	106 (60.9%)	68 (39.1%)		108 (69.1%)	66 (30.9%)		82 (47.1%)	92 (52.9%)	
Lymph node metastasis									
Negative (n = 136)	69 (50.7%)	67 (49.3%)	0.003^b^	79 (58.1%)	57 (41.9%)	0.159	49 (36.0%)	87 (64.0%)	0.001^b^
Positive (n = 89)	63 (70.8%)	26 (29.2%)		60 (67.4%)	29 (32.6%)		52 (58.4%)	37 (41.6%)	
Dukes’ stage									
A + B (n = 122)	63 (51.6%)	59 (48.4%)	0.020^b^	68 (55.7%)	54 (44.3%)	0.042^b^	43 (35.2%)	79 (64.8%)	0.002^b^
C + D (n = 103)	69 (67.0%)	34 (33.0%)		71 (68.9%)	32 (31.1%)		58 (56.3%)	45 (43.7%)	

^a^*P* value is obtained by Pearson chi-square, Asymp. Sig., two tailed

^b^*P* < 0.05 was designated as significance

### Activation of PKCδ induced B7-H4 expression in CRC cell lines

**Fig. 3:**
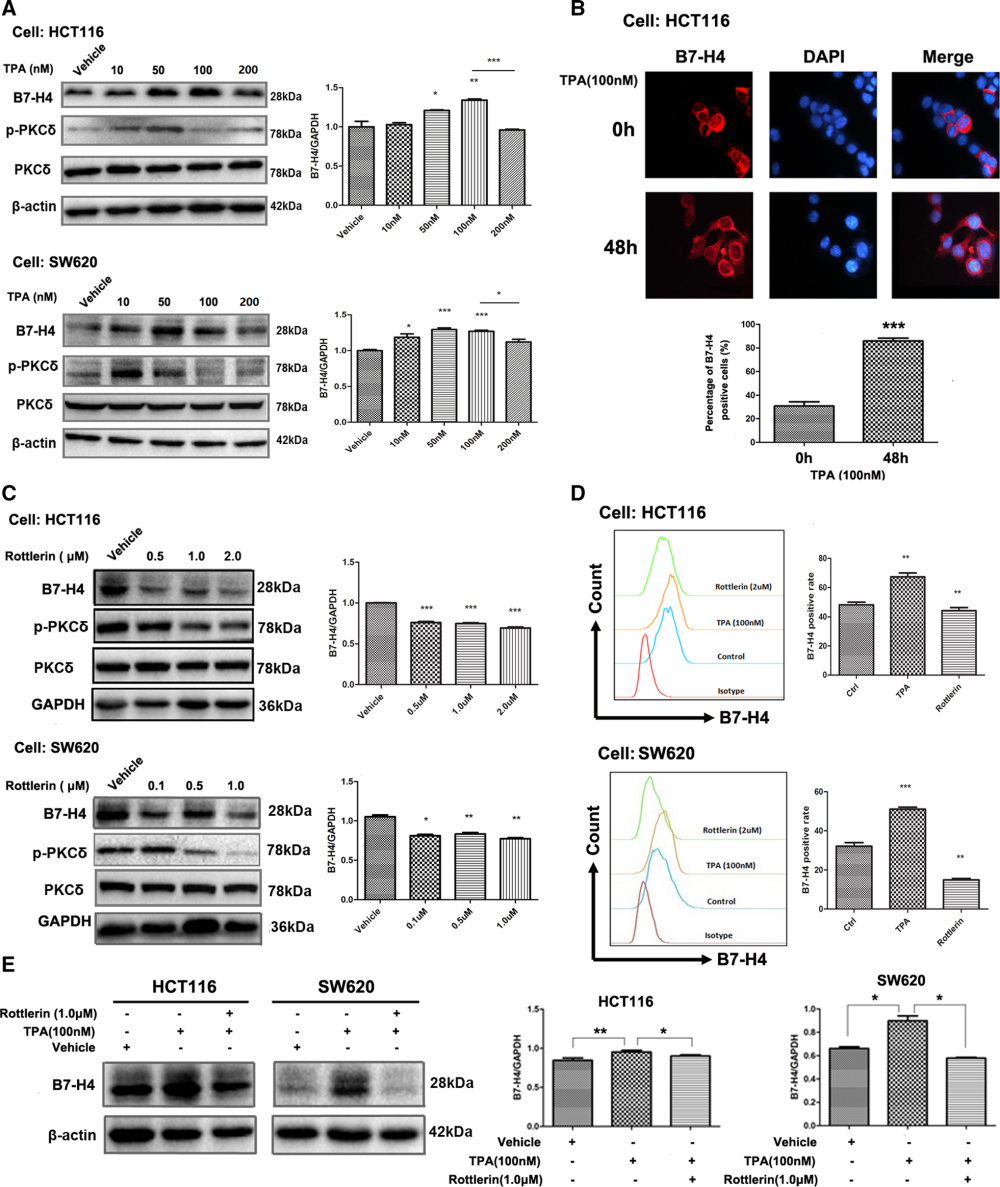
PKCδ mediates B7-H4 upregulation in CRC cell lines. HCT116 and SW620 cells were treated with various concentrations of TPA (**A**) or rottlerin (**C**) for 20 h. **B** B7-H4 and nuclei in HCT116 cells were stained with a PE-conjugated anti-B7-H4 antibody and DAPI, respectively. **D** Flow cytometric analysis was used to detect changes in B7-H4 expression in TPA- or rottlerin-treated HCT116 and SW620 cells. CRC cells were treated with TPA or rottlerin for 24 h, and intracellular B7-H4 expression was examined by flow cytometry. **E** The HCT116 and SW620 cell lines were treated with 1 μM rottlerin and 100 nM TPA for 24 h, and B7-H4 levels were determined by Western blotting. A representative Western blot image is shown in the left panel. Statistical analysis of B7-H4 expression (mean ± SD) from three separate experiments is shown in the right panel. *P < 0.05, **P < 0.01 and ***P < 0.001

Since TPA can induce strong PKCδ localization mainly to the plasma membrane [26], we examined the effect of TPA on B7-H4 expression in CRC cell lines. HCT116 and SW620 cells were treated with various concentrations of TPA for 20 h, and B7-H4 protein levels were determined by Western blotting. As shown in Fig. 3A and Additional file 3: Figure S3A, treatment with 10 and 50 nM TPA for 20 h effectively increased the p-PKCδ level, whereas only treatment with 50 nM TPA effectively increased the B7-H4 level. Treatment with 100 nM TPA for 20 h began to deactivate PKCδ, possibly due to the effect of chronic treatment with TPA [42, 43]. Similarly, time point at which B7-H4 expression began to decrease was delayed, and the required concentration increased to 200 nM. The explanation for the delay between these decreases in B7-H4 expression and PKCδ deactivation might be due to the time needed for signal transduction downstream of PKCδ. Therefore, the delay strongly confirmed that B7-H4 was regulated by the PKCδ signaling pathway. B7-H4 and nuclei in HCT116 cells were stained with a PE-conjugated anti-B7-H4 antibody and DAPI, respectively, and the cells were then observed under a confocal microscope (Fig. 3B). The results showed that TPA treatment significantly increased the percentage of B7-H4 positive cells. Furthermore, cell lines were treated with rottlerin, a PKCδ inhibitor. Rottlerin inhibited PKCα, PKCβ, PKCγ, PKCδ, PKCη, CKII and PKA and preferentially inhibited PKCδ activity at low concentrations but inhibited the other PKC isoforms at high concentrations[44]; thus, we used rottlerin at a concentration of 1–2 μM (low concentration) to investigate the inhibitory effect of PKCδ on B7-H4. The results showed that rottlerin effectively decreased the B7-H4 protein level in a concentration-dependent manner in the HCT116 and SW620 cell lines (Fig. 3C and Additional file 3: Figure S3B). Flow cytometric analysis also demonstrated that TPA increased B7-H4 expression and that rottlerin decreased B7-H4 expression in the HCT116 and SW620 cell lines (Fig. 3D). Then, the HCT116 and SW620 cell lines were treated first with 1 μM rottlerin and then with 100 nM TPA. This experiment showed that rottlerin effectively abrogated the TPA-induced increase in B7-H4 expression (Fig. 3E and Additional file 3: Figure S3C).

### PKCδ activation upregulated B7-H4 expression in CRC cell lines

**Fig. 4:**
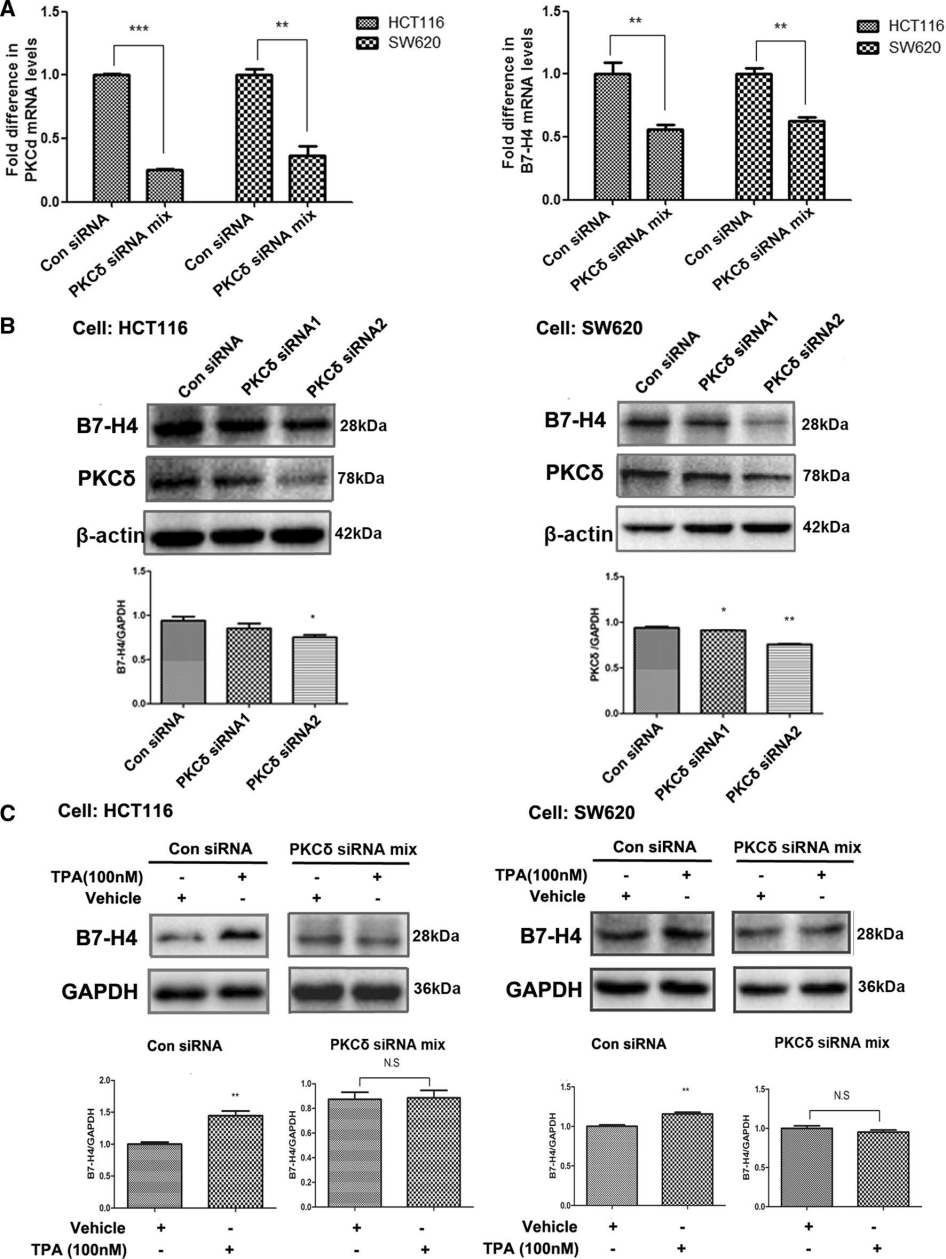
PKCδ knockdown inhibited the expression of B7-H4 in CRC cell lines. HCT116 and SW620 cells were treated with a PKCδ-specific siRNA for 45 h. B7-H4 and PKCδ levels were determined by quantitative RT–PCR (**A**) and Western blotting (**B**). HCT116 and SW620 cells were treated with a PKCδ-specific siRNA for 24 h and were then incubated with TPA (100 nM) for 20 h (**C**). The cells were harvested to generate whole-cell lysates for detection of the indicated proteins by Western blot analysis. A representative Western blot and B7-H4 expression data (mean ± SD) from three separate experiments are shown. *P < 0.05, **P < 0.01 and ***P < 0.001

After using a PKCδ activator and a PKCδ inhibitor, we knocked down PKCδ expression and examined the effect on B7-H4 expression in CRC cell lines. PKCδ mRNA expression was knocked down in HCT116 and SW620 cells as described in the Materials and Methods section. The PKCδ mRNA level was decreased dramatically in the treated group compared with the mock group (Fig. 4A). Western blot analysis showed that the PKCδ and B7-H4 protein levels were significantly decreased in the PKCδ siRNA/HCT116 and PKCδ siRNA/SW620 cell lines (Fig. 4B and Additional file 4: Figure S4A). The con siRNA and PKCδ siRNA cell lines were treated with TPA, and PKCδ knockdown was found to abrogate the TPA-induced increase in B7-H4 expression (Fig. 4C and Additional file 4: Figure S4B). Taken together, these data suggested that PKCδ knockdown downregulated B7-H4 expression in CRC cells.

### STAT3 mediated PKCδ-induced B7-H4 upregulation in CRC cell lines

**Fig. 5:**
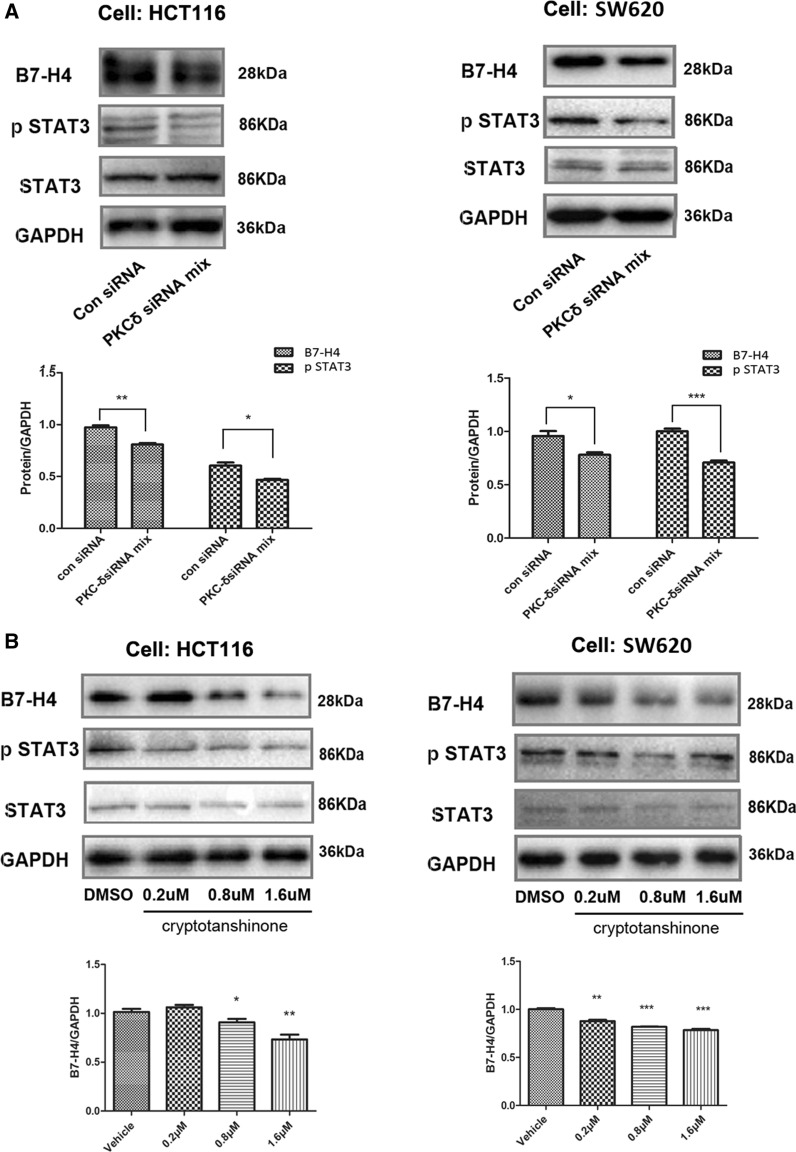
PKCδ inhibited B7-H4 expression via STAT3 in CRC cell lines. Treatment with a PKCδ-specific siRNA reduced the expression of both B7-H4 and STAT3 in HCT116 and SW620 cells (**A**). HCT116 and SW620 cells were treated with various concentrations of the STAT3 inhibitor cryptotanshinone (**B**) for 24 h. The cells were harvested to generate whole-cell lysates for detection of the indicated proteins by Western blot analysis. A representative Western blot and B7-H4 expression data (mean ± SD) from three separate experiments are shown. *P < 0.05, **P < 0.01 and ***P < 0.001

We next explored the signaling pathway by which PKCδ mediates the expression of B7-H4. A previous study showed that activated STAT3 can bind to the B7-H4 promoter and enhance the expression of the B7-H4 protein in microglial cells [45]. In addition, PKCδ is a primary regulator of STAT3 phosphorylation in keratinocytes and luteal cells [46, 47]. Therefore, we hypothesized that activated PKCδ can induce B7-H4 expression by increasing the phosphorylation of STAT3 in CRC cells. Western blot analysis showed that the levels of B7-H4 and p-STAT3 were decreased in PKCδ siRNA CRC cells (Fig. 5A and Additional file 5: Figure S5A). Moreover, we observed that cryptotanshinone, a STAT3 phosphorylation inhibitor, significantly decreased the B7-H4 protein level in a concentration-dependent manner in HCT116 and SW620 cells (Fig. 5B and Additional file 5: Figure S5B). These results suggested that PKCδ could regulate the expression of B7-H4 via the STAT3 signaling pathway in CRC cells.

### The PKCδ/B7-H4 axis promoted CRC cell motility

**Fig. 6:**
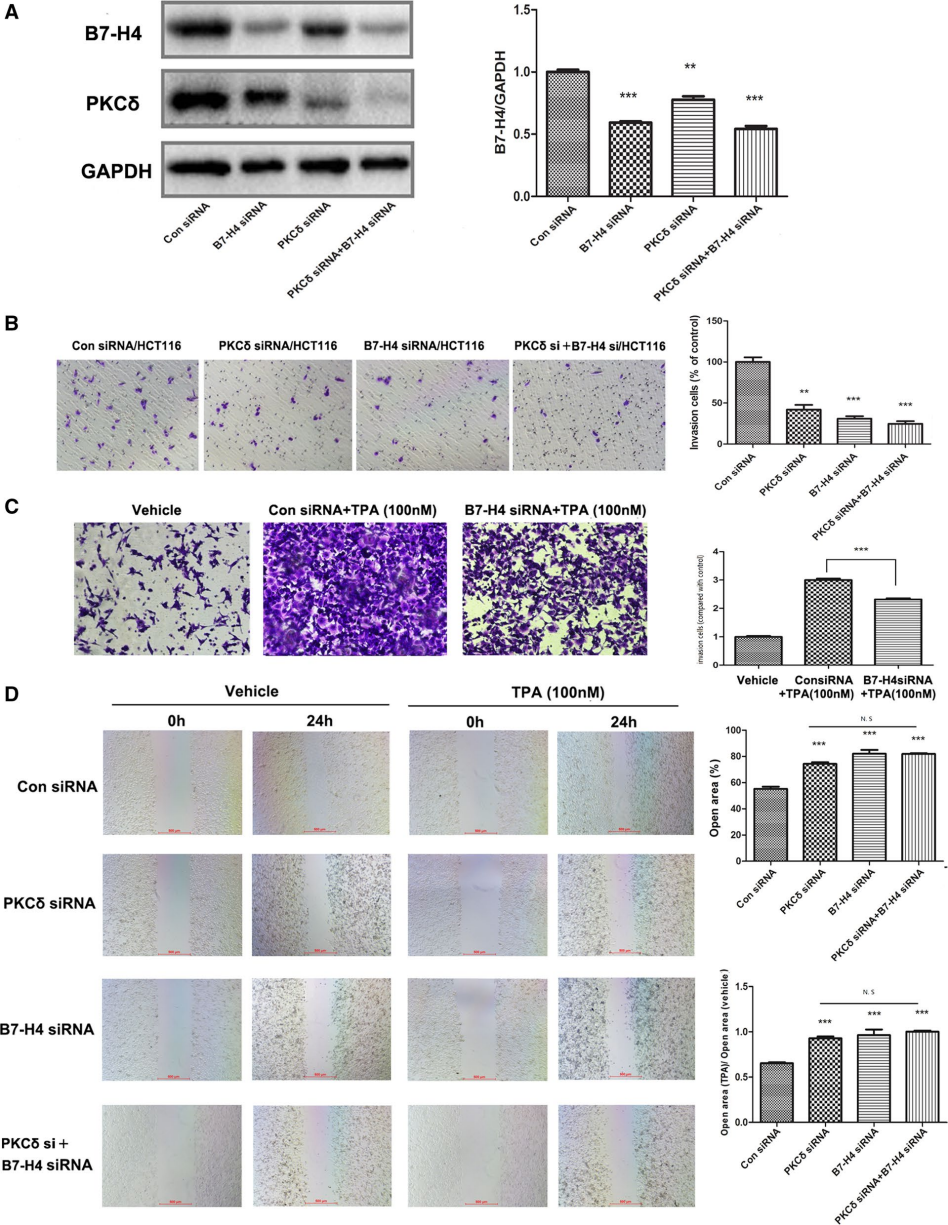
The PKCδ/B7-H4 axis promoted the migration of HCT116 cells. HCT116 cells were treated with a PKCδ-specific siRNA and/or a B7-H4-specific siRNA for 45 h, and B7-H4 protein levels were then determined by Western blot analysis (**A**). A Transwell assay was performed to examine the constitutive invasion of B7-H4 siRNA/HCT116, PKCδ siRNA/HCT116, PKCδ siRNA + B7-H4 siRNA/HCT116 and con siRNA/HCT116 cells (**B**). The invasion of B7-H4 siRNA/HCT116 cells was evaluated after the cells were treated with 100 nM TPA for 24 h (**C**). A wound healing assay was performed to evaluate the effects of PKCδ and B7-H4 on cell migration (**D**). Experiments were performed in triplicate. *P < 0.05, **P < 0.01 and ***P < 0.001

As analysis of clinical samples and datasets showed that B7-H4 and p-PKCδ were associated with CRC metastasis, we further examined whether CRC cell invasion and migration are mediated via the PKCδ/B7-H4 axis. Previously, the PKCδ siRNA/HCT116 and PKCδ siRNA/SW620 cell lines were established. Here, we established the B7-H4 siRNA/HCT116 and B7-H4 siRNA/SW620 cell lines (Fig. 6A and Additional file 6: Figure S6A). A Transwell assay showed that knockdown of B7-H4 or PKCδ expression inhibited the constitutive invasion of CRC cells (Fig. 6B and Additional file 6: Figure S6B), suggesting that B7-H4 and PKCδ promoted cell motility. In addition, we found that pharmacological induction of PKCδ expression by 24 h of TPA treatment effectively enhanced cell invasion (Fig. 6C). However, knockdown of B7-H4 prevented the increase in invasion (Fig. 6C), suggesting that B7-H4 plays a role in PKCδ activation-induced cell invasion.

To evaluate the effects of PKCδ and B7-H4 on cell migration, we performed a wound healing assay. Images showing wound closure are shown in Fig. 6D and Additional file 6: Figure S6B. Knockdown of PKCδ or B7-H4 expression effectively inhibited cell motility. TPA treatment effectively enhanced the motility of con siRNA/HCT116 cells, but the effect was obviously reduced in B7-H4 siRNA/HCT116 and PKCδ siRNA/HCT116 cells. Cell viability was also assessed by a wound healing assay. There were no differences between the TPA treatment groups and the control groups (data not shown).

### Rottlerin inhibited B7-H4 expression and tumor metastasis in mice

**Fig. 7:**
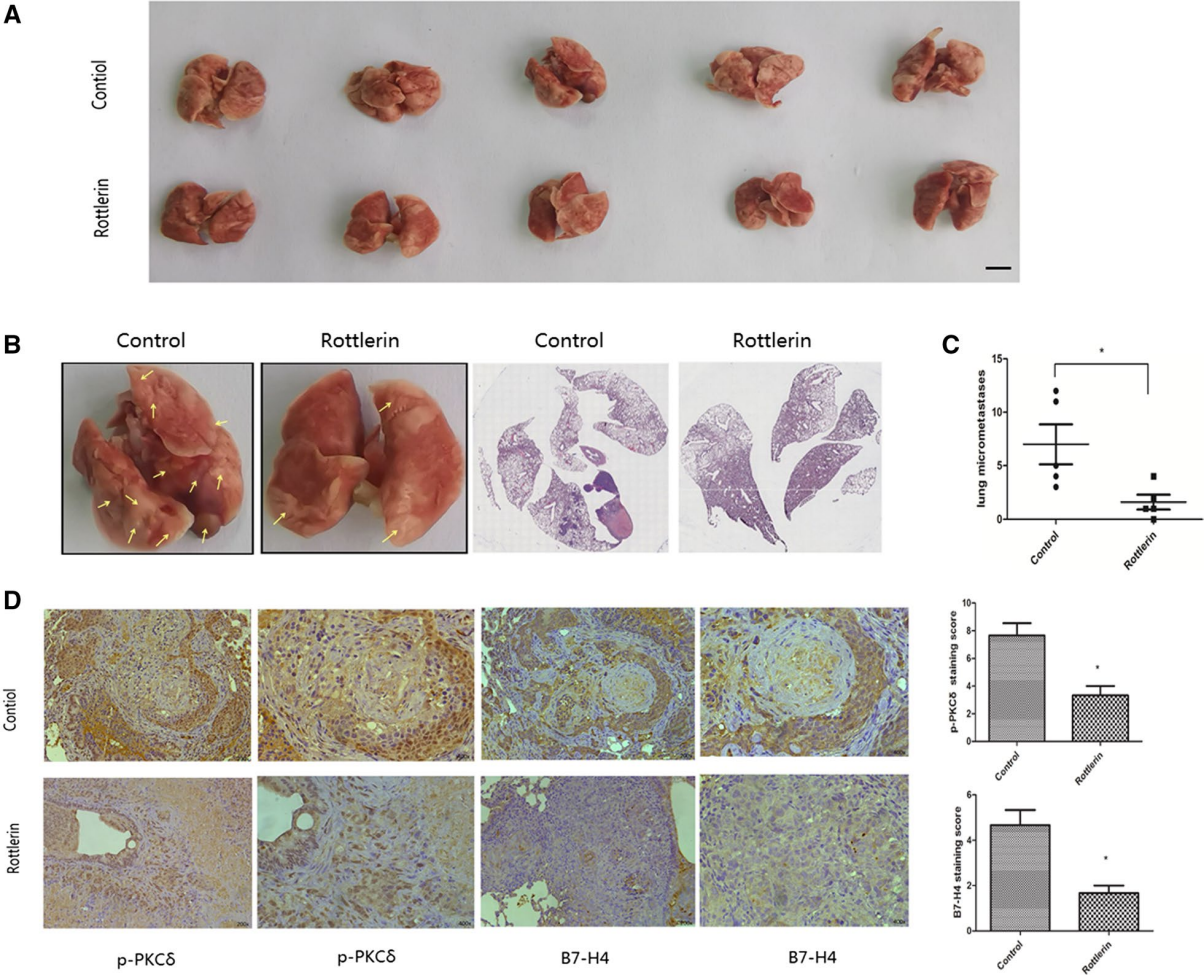
**A** Representative images of lung metastatic nodules developed in mice 54 days after injection of HCT116 cells. The isolated lungs were processed as described in the Materials and Methods section. Scale bar, 5 mm. **B**, **C** Representative images of lung metastatic nodules and H&E staining. The red arrowheads indicate metastatic nodules established in the lungs. H&E staining was performed to evaluate lung micrometastases in a pair of mice referred to in **A**. **D** Immunohistochemical analysis of p-PKCδ and B7-H4 in the two groups. The data are presented as the mean ± SD values (*n* = 5 animals/group). *P < 0.05; **P < 0.01

To validate the effect of the PKCδ/B7-H4 axis on tumor metastasis in vivo, we injected rottlerin into HCT116 tumor-bearing nude mice. Rottlerin treatment significantly reduced lung metastasis (Fig. 7A and B). The IHC results showed that the levels of human B7-H4 and p-PKCδ were decreased in the lung tissues of rottlerin-treated mice (Fig. 7C; lung tissues from three mice per group). These results show that rottlerin treatment inhibited colon cancer metastasis compared with that in the control group via the PKCδ/B7-H4 axis.

## Discussion

CRC cell migration and invasion are related to the occurrence of postsurgical metastasis and poor survival in CRC patients; thus, we need to find new CRC therapies to block metastasis. As a novel member of the PKC family, PKCδ can be activated independent of Ca^2+^ and phospholipids and has multiple functions associated with cancer progression, including functions in the proliferation, survival, apoptosis and motility of cancer cells [30]. PKCδ is involved in colon epithelial cell migration via the IGF-I signaling pathway [34]. Although some studies found that PKCδ mRNA expression was decreased in most primary CRC tumors and some CRC cell lines, the p-PKCδ level was increased in a subset of colorectal cancers, and this increase can also enhance the migration and invasiveness of colon carcinoma cells by enhancing KITENIN expression and phosphorylation of HuR and Trop-2 [26, 27, 36]. Knockdown of B7-H4 effectively inhibits the proliferation, invasion, and migration of CRC cells, gastric cancer cells and lung cancer cells via various signaling pathways [20, 21, 48]. Both PKCδ and B7-H4 contribute to tumor metastasis; therefore, we sought to investigate the correlation among PKCδ, B7-H4 and metastasis in CRC.

In this study, we evaluated the protein levels of p-PKCδ and B7-H4 in tumor cells and found increased levels of p-PKCδ in some colorectal tumor samples (139/225, 61.8%). IHC analysis of serial sections from identical tissues showed that 101 of the p-PKCδ^+^ samples also expressed B7-H4 (72.7%, 101/139), and the IF staining results further confirmed this finding. The IHC results showed that a p-PKCδ^+^B7-H4^+^ phenotype in colorectal tumor samples was significantly associated with moderate/poor differentiation, lymph node metastasis and advanced Dukes’ stage. Thus, we speculated that the magnitude of PKCδ activation is related to the B7-H4 level and plays an important role in cancer progression.

It has been reported that B7-H4 expression can be upregulated by many inflammatory mediators. In a renal cell carcinoma cell line, IFN-α, IL-2, and IFN-γ were found to upregulate B7-H4 expression [49]. In human ovarian cancer and glioma cancer, tumor-associated Tregs trigger macrophages to secrete IL-10 and IL-6, which activate STAT3 and induce B7-H4 transcription [50, 51]. In human lung cancer, tumor-associated macrophages secrete TNF-α, IL-10, and IFN-γ, which induce B7-H4 expression in lung cancer cells [52]. Our previous study revealed that B7-H4 can be upregulated by IGF1R activation through the MEK/ERK1/2 signaling pathway in lung cancer [53]. In multiple myeloma, hypoxia-inducible factor-1α (HIF-1α) can bind to the B7-H4 promoter and induce B7-H4 expression [54]. In addition, the NF-κB (P65) pathway could increase PD-L1 and B7-H4 levels in hepatocellular carcinoma (HCC) tissues [55]. A recent study demonstrated that TGF-β1-driven SMAD3/4 signaling can increase B7-H4 expression in CRC [56]. Most of these studies identified the triggering factors contributing to B7-H4 expression, but the exact signaling pathways involved in the regulation of B7-H4 expression still require further elucidation.

In this study, we found that the protein levels of p-PKCδ and B7-H4 were higher in CRC cell lines than in a normal cell line. The PKC activator TPA increased the B7-H4 level in HCT116 and SW620 cells in a concentration-dependent manner. To confirm that B7-H4 is regulated by activation of PKCδ, the specific PKCδ inhibitor rottlerin and a PKCδ-specific siRNA were used. Initially, rottlerin was found to inhibit PKC partially due to competition with ATP for the ATP-binding site in PKC, and the structure of rottlerin is much more selective for the PKCδ isozyme [44]. However, a series of subsequent studies showed that rottlerin can inhibit agonist-induced PKCδ translocation, thereby inhibiting PKCδ activity [57, 58]. Regardless of the exact mechanism, all of these studies have shown that rottlerin can selectively inhibit PKCδ activation. Therefore, in this study, we used rottlerin as a specific PKCδ inhibitor. The results showed that rottlerin decreased B7-H4 expression and abrogated the TPA-induced increase in B7-H4 expression. Furthermore, treatment with the PKCδ-specific siRNA also effectively decreased the B7-H4 level and abrogated the TPA-induced increase in B7-H4 expression. Collectively, these results verified the findings reported for clinical samples and confirmed that B7-H4 expression can be upregulated by PKCδ activation. Moreover, PKCδ siRNA treatment also reduced the activity of STAT3. Treatment with the STAT3 inhibitor cryptotanshinone significantly decreased the B7-H4 protein level in a concentration-dependent manner in CRC cell lines. These results suggested that PKCδ could regulate the expression of B7-H4 via STAT3. We also investigated whether PKCδ promotes B7-H4 expression through the MEK/ERK1/2 pathway in CRC cells, but we did not find a related change (data not shown). Previous studies revealed that PKCδ plays an important role in colon cancer cell migration and invasion. B7-H4 could increase cell migration and invasion by targeting the angiogenic factors MMP2, MMP9 and VEGF. B7-H4 overexpression activates a variety of signaling pathways, such as the NF-κB, ERK1/2, AKT/STAT3 and PI3K/AKT/mTOR pathways, to promote epithelial-mesenchymal transition (EMT) and invasion of cancer cells [20, 22, 59, 60]. Here, we also performed Transwell invasion and wound healing assays to evaluate the motility of CRC cells. We found that knockdown of B7-H4 or PKCδ suppressed cell motility and suppressed the enhancing effect of TPA on cell invasion and migration. We also found that rottlerin treatment significantly inhibited B7-H4 expression and tumor metastasis in vivo. The rottlerin dose (20 mg/kg) used in the present study was lower than the safe dose established in a previous study.

In conclusion, our results identify a canonical PKCδ/STAT3/B7-H4 signaling pathway that is constitutively active in colorectal carcinoma cells. B7-H4 expression was upregulated by PKCδ activation and contributed to PKCδ-induced cell motility, which plays a role in the immune escape of CSCs. This result suggests that B7-H4 and PKCδ may be therapeutic targets in tumor metastasis. It might be important to consider the effect on B7-H4 expression when a PKCδ inhibitor is used clinically.

## Supplementary Information

**Additional file 1: Figure S1.** Correlation analysis of the expression of PKCs and B7-H4 in CRC based on the LinkedOmics and GEPIA databases. **A** Correlation analysis between PKCs and B7-H4; **B** comparison of PKRCA and PRKCD expression in cancer and adjacent tissues.

**Additional file 2: Figure S2.** Western blot analysis was performed to detect the expression of B7-H4 in CRC cell lines. The protein levels of B7-H4 and p-PKCδ in the NCM460, SW480, HCT116, SW620 and RKO cell lines were determined.

**Additional file 3: Figure S3.** PKCδ mediated B7-H4 upregulation in CRC cell lines. HCT116 and SW620 cells were treated with various concentrations of TPA (**A**) or rottlerin (**B**) for 20 h. **C** The HCT116 and SW620 cell lines were treated with 1 μM rottlerin and 100 nM TPA for 24 h, and B7-H4 levels were determined by Western blotting.

**Additional file 4: Figure S4.** PKCδ knockdown inhibited the expression of B7-H4 in CRC cell lines. HCT116 and SW620 cells were treated with a PKCδ-specific siRNA for 45 h. B7-H4 and PKCδ levels were determined by Western blotting (**A**). HCT116 and SW620 cells were treated with a PKCδ-specific siRNA for 24 h and were then incubated with TPA (100 nM) for 20 h (**B**, **C**). The cells were harvested to generate whole-cell lysates for detection of the indicated proteins by Western blot analysis.

**Additional file 5: Figure S5.** PKCδ inhibited B7-H4 expression via STAT3 in CRC cell lines. Treatment with a PKCδ-specific siRNA reduced the expression of both B7-H4 and STAT3 in HCT116 and SW620 cells (**A**). HCT116 and SW620 cells were treated with various concentrations of the STAT3 inhibitor cryptotanshinone (**B**) for 24 h. The cells were harvested to generate whole-cell lysates for detection of the indicated proteins by Western blot analysis.

**Additional file 6: Figure S6.** The PKCδ/B7-H4 axis promoted the migration of SW620 cells. HCT116 and SW620 cells were treated with a PKCδ-specific siRNA and/or a B7-H4-specific siRNA for 45 h, and B7-H4 protein levels were then determined by Western blot analysis (**A**). A Transwell assay was performed to examine the constitutive invasion of B7-H4 siRNA/SW620, PKCδ siRNA/SW620, PKCδ siRNA + B7-H4 siRNA/SW620 and con siRNA/SW620 cells (**B**). A wound healing assay was performed to evaluate the effects of PKCδ and B7-H4 on cell migration (**C**). The viability of HCT116 cells in different groups was assessed by a CCK-8 assay (**D**). Experiments were performed in triplicate. *P < 0.05, **P < 0.01 and ***P < 0.001.

**Additional file 7: Table S1.** The primers of real-time PCR and the siRNAs.

## Data Availability

The datasets generated during the current study can be discovered in online repositories, further inquiries can be available from the corresponding author.

## Abbreviations

CRC: Colorectal cancer
PKC: Protein kinase C
TPA: Phorbol-12-Myristate-13-Acetate
APCs: Antigen-presenting cells
Treg: Regulatory T cells
siRNA: Small interfering RNA
IHC: Immunohistochemistry
IF: Immunofluorescence
FBS: Fetal bovine serum
SDS-PAGE: Sodium dodecyl sulfate–polyacrylamide gel
PVDF: Polyvinylidene difluoride
FCS: Flow cytometry analysis
HIF-1α: Hypoxia-inducible factor-1α
HCC: Hepatocellular carcinoma

## References

[1] Morris EJ, Forman D, Thomas JD, Quirke P, Taylor EF, Fairley L (2010). Surgical management and outcomes of colorectal cancer liver metastases. Br J Surg.

[2] Xu G, Zhou Y, Zhou F (2018). Development and validation of an immunity-related classifier of nine chemokines for predicting recurrence in stage I–III patients with colorectal cancer after operation. Cancer Manag Res.

[3] Levin B, Lieberman DA, McFarland B, Andrews KS, Brooks D, Bond J (2008). Screening and surveillance for the early detection of colorectal cancer and adenomatous polyps, 2008: a joint guideline from the American Cancer Society, the US Multi-Society Task Force on Colorectal Cancer, and the American College of Radiology. Gastroenterology.

[4] Han EC, Kwon Y-H, Park KJ, Jeong S-Y, Kang S-B, Oh JH (2018). Significance of lymph node metastasis in the survival of stage IV colorectal cancer by hematogenous metastasis. Ann Surg Treat Res.

[5] Zang X, Loke P, Kim J, Murphy K, Waitz R, Allison JP (2003). B7x: a widely expressed B7 family member that inhibits T cell activation. Proc Natl Acad Sci USA.

[6] Sica GL, Choi IH, Zhu G, Tamada K, Wang SD, Tamura H (2003). B7–H4, a molecule of the B7 family, negatively regulates T cell immunity. Immunity.

[7] He C, Qiao H, Jiang H, Sun X (2011). The inhibitory role of b7–h4 in antitumor immunity: association with cancer progression and survival. Clin Dev Immunol..

[8] Krambeck AE, Thompson RH, Dong H, Lohse CM, Park ES, Kuntz SM (2006). B7–H4 expression in renal cell carcinoma and tumor vasculature: associations with cancer progression and survival. Proc Natl Acad Sci USA.

[9] Zang X, Thompson RH, Al-Ahmadie HA, Serio AM, Reuter VE, Eastham JA (2007). B7–H3 and B7x are highly expressed in human prostate cancer and associated with disease spread and poor outcome. Proc Natl Acad Sci USA.

[10] Assal A, Kaner J, Pendurti G, Zang X (2015). Emerging targets in cancer immunotherapy: beyond CTLA-4 and PD-1. Immunotherapy.

[11] John P, Wei Y, Liu W, Du M, Guan F, Zang X (2019). The B7x immune checkpoint pathway: from discovery to clinical trial. Trends Pharmacol Sci.

[12] Podojil JR, Glaser AP, Baker D, Courtois ET, Fantini D, Yu Y (2020). Antibody targeting of B7–H4 enhances the immune response in urothelial carcinoma. Oncoimmunology.

[13] Yan JFX, Hong B, Qian Y (2021). The expression of PD-L1 and B7–H4 in thymic epithelial tumor and its relationship with tumor immune-infiltrating cells. Front Oncol.

[14] Zhao LW, Li C, Zhang RL, Xue HG, Zhang FX, Zhang F (2014). B7–H1 and B7–H4 expression in colorectal carcinoma: correlation with tumor FOXP3(+) regulatory T-cell infiltration. Acta Histochem.

[15] Cao H, Wang Q, Gao Z, Xu X, Lu Q, Wu Y (2019). Clinical value of detecting IQGAP3, B7–H4 and cyclooxygenase-2 in the diagnosis and prognostic evaluation of colorectal cancer. Cancer Cell Int.

[16] Simon I, Zhuo S, Corral L, Diamandis EP, Sarno MJ, Wolfert RL (2006). B7–h4 is a novel membrane-bound protein and a candidate serum and tissue biomarker for ovarian cancer. Cancer Res.

[17] Thompson RH, Zang X, Lohse CM, Leibovich BC, Slovin SF, Reuter VE (2008). Serum-soluble B7x is elevated in renal cell carcinoma patients and is associated with advanced stage. Can Res.

[18] Xu CH, Wang W, Wang YC, Lin Y, Zhang XW (2018). Diagnosis value of serum soluble B7–H4 expression in non-small cell lung cancer. Clin Respir J.

[19] Wang P, Li C, Zhang F, Ma X, Gai X (2018). Clinical value of combined determination of serum B7–H4 with carcinoembryonic antigen, osteopontin, or tissue polypeptide-specific antigen for the diagnosis of colorectal cancer. Dis Markers.

[20] Li C, Zhan Y, Ma X, Fang H, Gai X (2020). B7–H4 facilitates proliferation and metastasis of colorectal carcinoma cell through PI3K/Akt/mTOR signaling pathway. Clin Exp Med.

[21] Zhang X, Cai L, Zhang G, Shen Y, Huang J (2017). B7–H4 promotes tumor growth and metastatic progression in lung cancer by impacting cell proliferation and survival. Oncotarget.

[22] Nan-Xie J-BC, Zhang L, Zhang P-F, Shen Y-H, Yang X (2017). Upregulation of B7–H4 promotes tumor progression of intrahepatic cholangiocarcinoma. Cell Death Dis.

[23] Nishizuka Y (1984). The role of protein kinase C in cell surface signal transduction and tumour promotion. Nature.

[24] Rosse C, Linch M, Kermorgant S, Cameron AJ, Boeckeler K, Parker PJ (2010). PKC and the control of localized signal dynamics. Nat Rev Mol Cell Biol.

[25] Pongracz J, Clark P, Neoptolemos JP, Lord JM (1995). Expression of protein kinase C isoenzymes in colorectal cancer tissue and their differential activation by different bile acids. Int J Cancer.

[26] Doller A, Winkler C, Azrilian I, Schulz S, Hartmann S, Pfeilschifter J (2011). High-constitutive HuR phosphorylation at Ser 318 by PKC{delta} propagates tumor relevant functions in colon carcinoma cells. Carcinogenesis.

[27] Park M, Kim WK, Song M, Park M, Kim H, Nam HJ (2013). Protein kinase C-delta-mediated recycling of active KIT in colon cancer. Clin Cancer Res.

[28] Qvit N, Mochly-Rosen D (2014). The many hats of protein kinase Cdelta: one enzyme with many functions. Biochem Soc Trans.

[29] Chen Z, Forman LW, Williams RM, Faller DV (2014). Protein kinase C-delta inactivation inhibits the proliferation and survival of cancer stem cells in culture and in vivo. BMC Cancer.

[30] Isakov N (2018). Protein kinase C (PKC) isoforms in cancer, tumor promotion and tumor suppression. Semin Cancer Biol.

[31] Reyland ME, Jones DNM (2016). Multifunctional roles of PKCδ: opportunities for targeted therapy in human disease. Pharmacol Ther.

[32] Hernández-Maqueda JG, Luna-Ulloa LB, Santoyo-Ramos P, Castañeda-Patlán MC, Robles-Flores M (2013). Protein kinase C delta negatively modulates canonical Wnt pathway and cell proliferation in colon tumor cell lines. PLoS ONE.

[33] Cerda SR, Bissonnette M, Scaglione-Sewell B, Lyons MR, Khare S, Mustafi R (2001). PKC-delta inhibits anchorage-dependent and -independent growth, enhances differentiation, and increases apoptosis in CaCo-2 cells. Gastroenterology.

[34] André F, Rigot V, Remacle-Bonnet M, Luis J, Pommier G, Marvaldi J (1999). Protein kinases C-gamma and -delta are involved in insulin-like growth factor I-induced migration of colonic epithelial cells. Gastroenterology.

[35] Kho DH, Bae JA, Lee JH, Cho HJ, Cho SH, Lee JH (2009). KITENIN recruits Dishevelled/PKC delta to form a functional complex and controls the migration and invasiveness of colorectal cancer cells. Gut.

[36] Mori Y, Akita K, Ojima K, Iwamoto S, Yamashita T, Morii E (2019). Trophoblast cell surface antigen 2 (Trop-2) phosphorylation by protein kinase C alpha/delta (PKCalpha/delta) enhances cell motility. J Biol Chem.

[37] Zhang L, Wu H, Lu D, Li G, Sun C, Song H (2013). The costimulatory molecule B7–H4 promote tumor progression and cell proliferation through translocating into nucleus. Oncogene.

[38] Bin Z, Guangbo Z, Yan G, Huan Z, Desheng L, Xueguang Z (2014). Overexpression of B7–H3 in CD133+ colorectal cancer cells is associated with cancer progression and survival in human patients. J Surg Res.

[39] Ohno I, Eibl G, Odinokova I, Edderkaoui M, Damoiseaux RD, Yazbec M, Abrol R (2010). Rottlerin stimulates apoptosis in pancreatic cancer cells through interactions with proteins of the Bcl-2 family. Am J Physiol Gastrointest Liver Physiol.

[40] Goldklang MP, Perez-Zoghbi JF, Trischler J, Nkyimbeng T (2013). Treatment of experimental asthma using a single small molecule with anti-inflammatory and BK channel-activating properties. FASEB J.

[41] Hao R-T, Zheng C, Wu C-Y, Xia E-J, Zhou X-F, Quan R-D, Zhang X-H (2019). NECTIN4 promotes papillary thyroid cancer cell proliferation, migration, and invasion and triggers EMT by activating AKT [Corrigendum]. Cancer Manag Res.

[42] Cartee L, Kucera GL, Nixon JB (1998). The effects of gemcitabine and TPA on PKC signaling in BG-1 human ovarian cancer cells. Oncol Res.

[43] Ohuchi T, Kuwaki T, Ling GY, Dewit D, Ju KH, Onodera M (1999). Elevation of blood pressure by genetic and pharmacological disruption of the ETB receptor in mice. Am J Physiol.

[44] Gschwendt M, Muller HJ, Kielbassa K, Zang R, Kittstein W, Rincke G, Marks F (1994). Rottlerin, a novel protein kinase inhibitor. Biochem Biophys Res Commun.

[45] Yao Y, Ye H, Qi Z, Mo L, Yue Q, Baral A (2016). B7–H4(B7x)-mediated cross-talk between glioma-initiating cells and macrophages via the IL6/JAK/STAT3 pathway lead to poor prognosis in glioma patients. Clin Cancer Res.

[46] Peters CA, Maizels ET, Robertson MC, Shiu RP, Soloff MS, Hunzicker-Dunn M (2000). Induction of relaxin messenger RNA expression in response to prolactin receptor activation requires protein kinase C delta signaling. Mol Endocrinol.

[47] Gartsbein M, Alt A, Hashimoto K, Nakajima K, Kuroki T, Tennenbaum T (2006). The role of protein kinase C delta activation and STAT3 Ser727 phosphorylation in insulin-induced keratinocyte proliferation. J Cell Sci.

[48] Zhou D, Zhou Y, Li C, Yang L (2018). Silencing of B7–H4 suppresses the tumorigenicity of the MGC-803 human gastric cancer cell line and promotes cell apoptosis via the mitochondrial signaling pathway. Int J Oncol.

[49] Xu Y, Zhu S, Song M, Liu W, Liu C, Li Y (2014). B7–H4 expression and its role in interleukin-2/interferon treatment of clear cell renal cell carcinoma. Oncol Lett.

[50] Kryczek I, Zou L, Rodriguez P, Zhu G, Wei S, Mottram P (2006). B7–H4 expression identifies a novel suppressive macrophage population in human ovarian carcinoma. J Exp Med.

[51] Kryczek I, Wei S, Zhu G, Myers L, Mottram P, Cheng P (2007). Relationship between B7–H4, regulatory T cells, and patient outcome in human ovarian carcinoma. Cancer Res.

[52] Chen C, Qu QX, Shen Y, Mu CY, Zhu YB, Zhang XG (2012). Induced expression of B7–H4 on the surface of lung cancer cell by the tumor-associated macrophages: a potential mechanism of immune escape. Cancer Lett.

[53] Zhao Z, Zhang N, Li A, Zhou B, Chen Y, Chen S (2020). Insulin-like growth factor-1 receptor induces immunosuppression in lung cancer by upregulating B7–H4 expression through the MEK/ERK signaling pathway. Cancer Lett.

[54] Jeon YK, Park S, Choi IW, Lee SW, Lee SM, Choi I (2015). Cancer cell-associated cytoplasmic B7–H4 is induced by hypoxia through hypoxia-inducible factor-1α and promotes cancer cell proliferation. Biochem Biophys Res Commun.

[55] Li QT, Qiu MJ, Yang SL, Fang X, He XX, Wang MM (2020). Alpha-fetoprotein regulates the expression of immune-related proteins through the NF-kappaB (P65) pathway in hepatocellular carcinoma cells. J Oncol.

[56] Zhou X, Mao Y, Zhu J, Meng F, Chen Q, Tao L (2016). TGF-beta1 promotes colorectal cancer immune escape by elevating B7–H3 and B7–H4 via the miR-155/miR-143 axis. Oncotarget.

[57] Park SW, Schonhoff CM, Webster CRL, Anwer MS (2012). Protein kinase Cδ differentially regulates cAMP-dependent translocation of NTCP and MRP2 to the plasma membrane. Am J Physiol Gastrointest Liver Physiol.

[58] Lee T-H, Chen J-L, Liu P-S, Tsa M-M, Wang S-J, Hsieh H-L (2020). Rottlerin, a natural polyphenol compound, inhibits upregulation of matrix metalloproteinase-9 and brain astrocytic migration by reducing PKC-δ-dependent ROS signal. J Neuroinflammation.

[59] Chen L, Jin M, Li C, Shang Y, Zhang Q (2017). The tissue distribution and significance of B7–H4 in laryngeal carcinoma. Oncotarget.

[60] Wu H, Wang X, Mo N, Zhang L, Yuan X, Lu Z (2018). B7-homolog 4 promotes epithelial-mesenchymal transition and invasion of bladder cancer cells via activation of nuclear factor-kappaB. Oncol Res.

