# Supplementary figures and images for: B7-H4 expression is upregulated by PKCδ activation and contributes to PKCδ-induced cell motility in colorectal cancer
Source: Cancer Cell Int. 2022 Apr 11;22:147. doi: 10.1186/s12935-022-02567-1 (PMC8996430; doi:10.1186/s12935-022-02567-1)

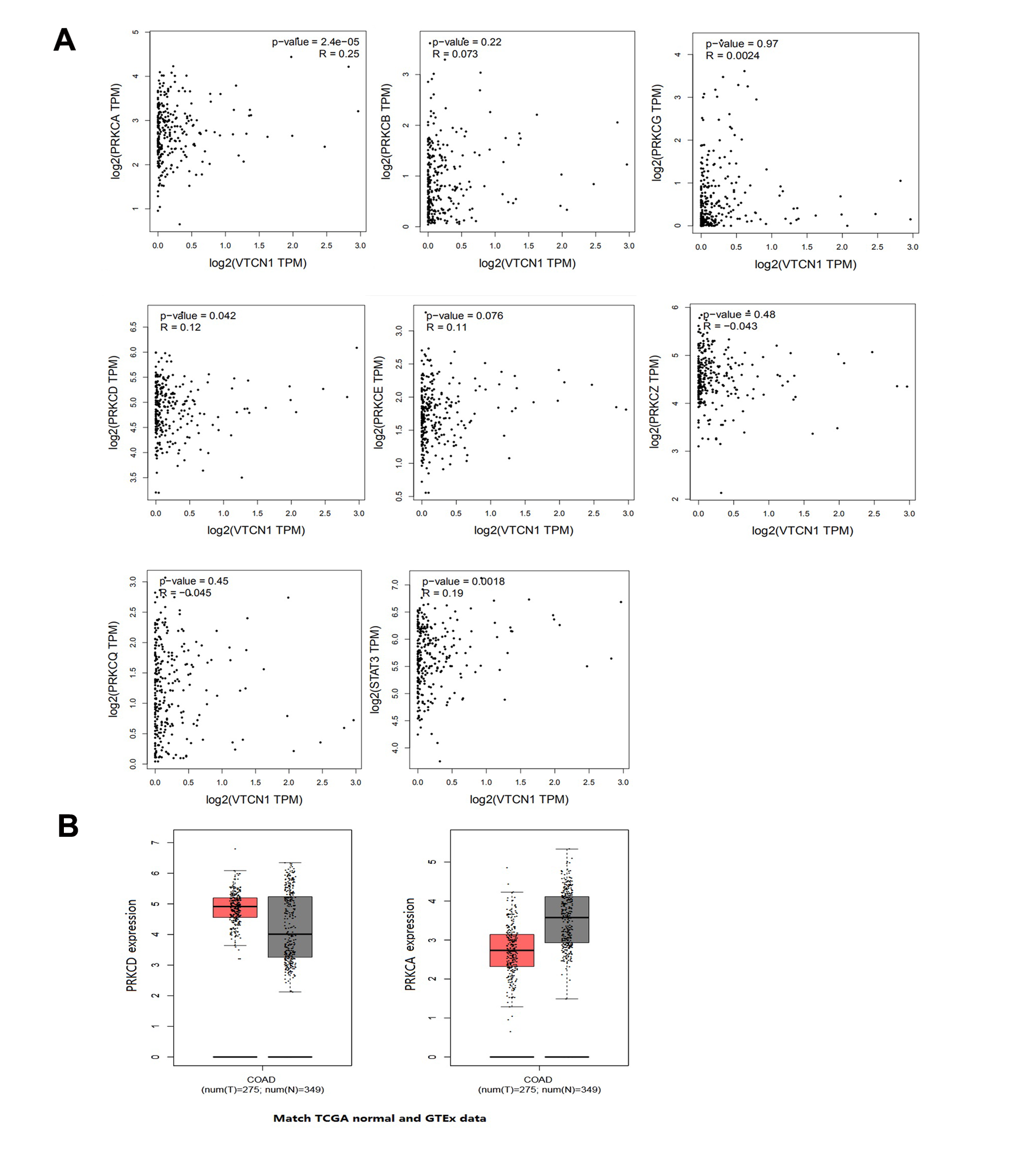

Supplement: Supplementary file 1 — Additional file 1: Figure S1. Correlation analysis of the expression of PKCs and B7-H4 in CRC based on the LinkedOmics and GEPIA databases. A Correlation analysis between PKCs and B7-H4; B comparison of PKRCA and PRKCD expression in cancer and adjacent tissues. [file 12935_2022_2567_MOESM1_ESM.tif]

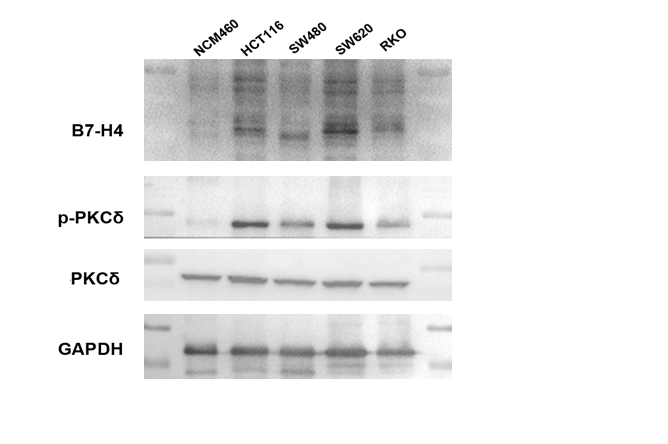

Supplement: Supplementary file 2 — Additional file 2: Figure S2. Western blot analysis was performed to detect the expression of B7-H4 in CRC cell lines. The protein levels of B7-H4 and p-PKCδ in the NCM460, SW480, HCT116, SW620 and RKO cell lines were determined. [file 12935_2022_2567_MOESM2_ESM.tif]

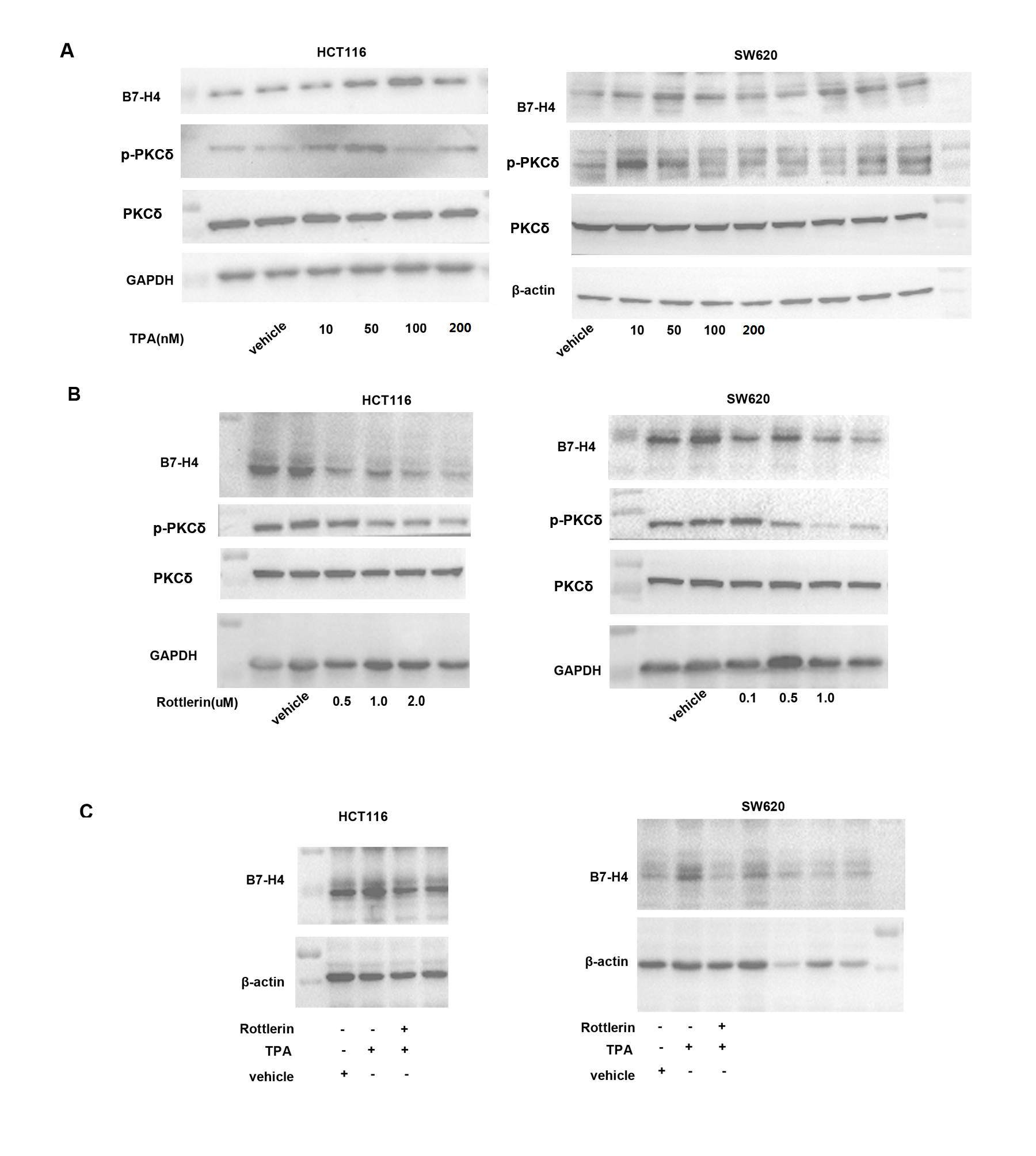

Supplement: Supplementary file 3 — Additional file 3: Figure S3. PKCδ mediated B7-H4 upregulation in CRC cell lines. HCT116 and SW620 cells were treated with various concentrations of TPA (A) or rottlerin (B) for 20 h. C The HCT116 and SW620 cell lines were treated with 1 μM rottlerin and 100 nM TPA for 24 h, and B7-H4 levels were determined by Western blotting. [file 12935_2022_2567_MOESM3_ESM.tif]

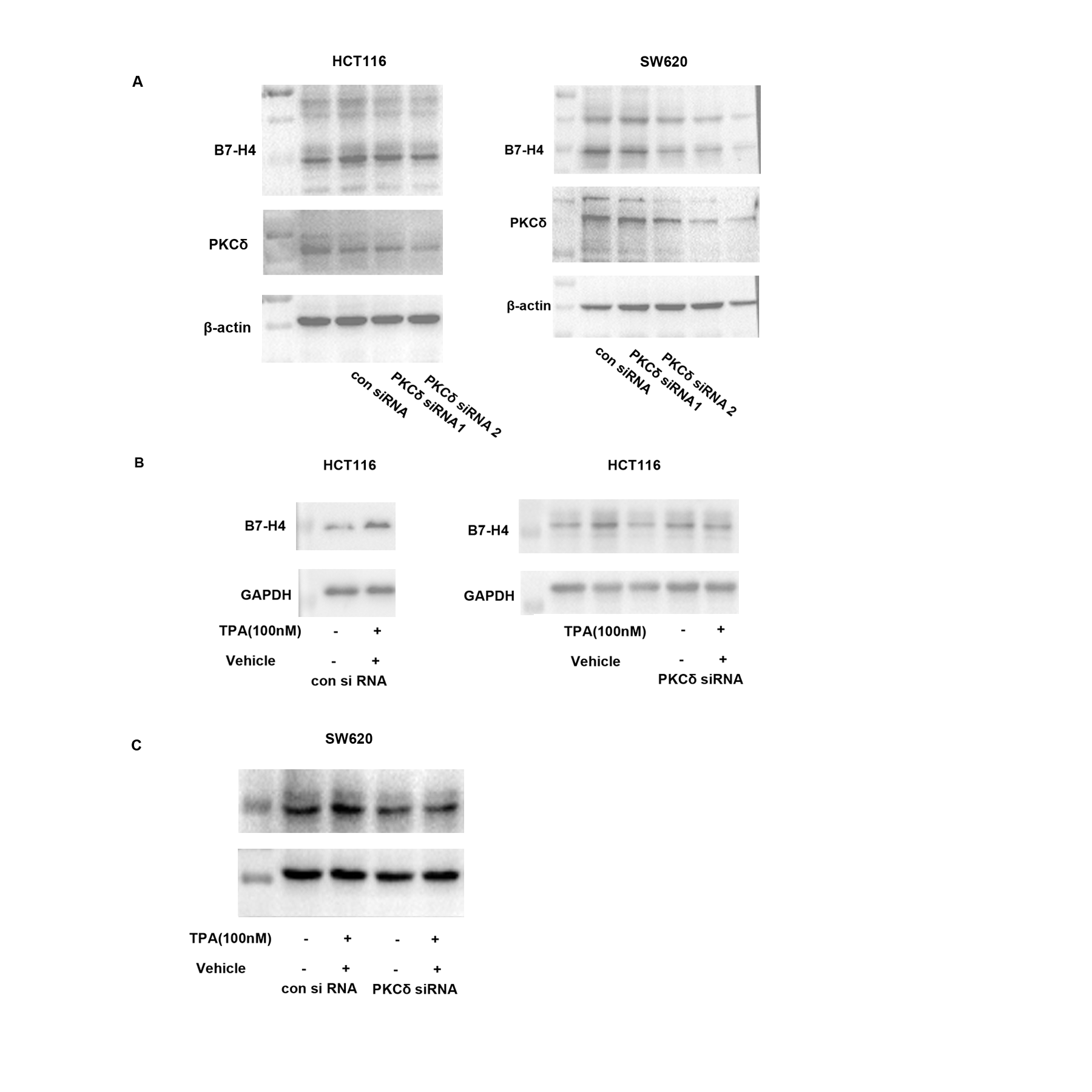

Supplement: Supplementary file 4 — Additional file 4: Figure S4. PKCδ knockdown inhibited the expression of B7-H4 in CRC cell lines. HCT116 and SW620 cells were treated with a PKCδ-specific siRNA for 45 h. B7-H4 and PKCδ levels were determined by Western blotting (A). HCT116 and SW620 cells were treated with a PKCδ-specific siRNA for 24 h and were then incubated with TPA (100 nM) for 20 h (B, C). The cells were harvested to generate whole-cell lysates for detection of the indicated proteins by Western blot analysis. [file 12935_2022_2567_MOESM4_ESM.tif]

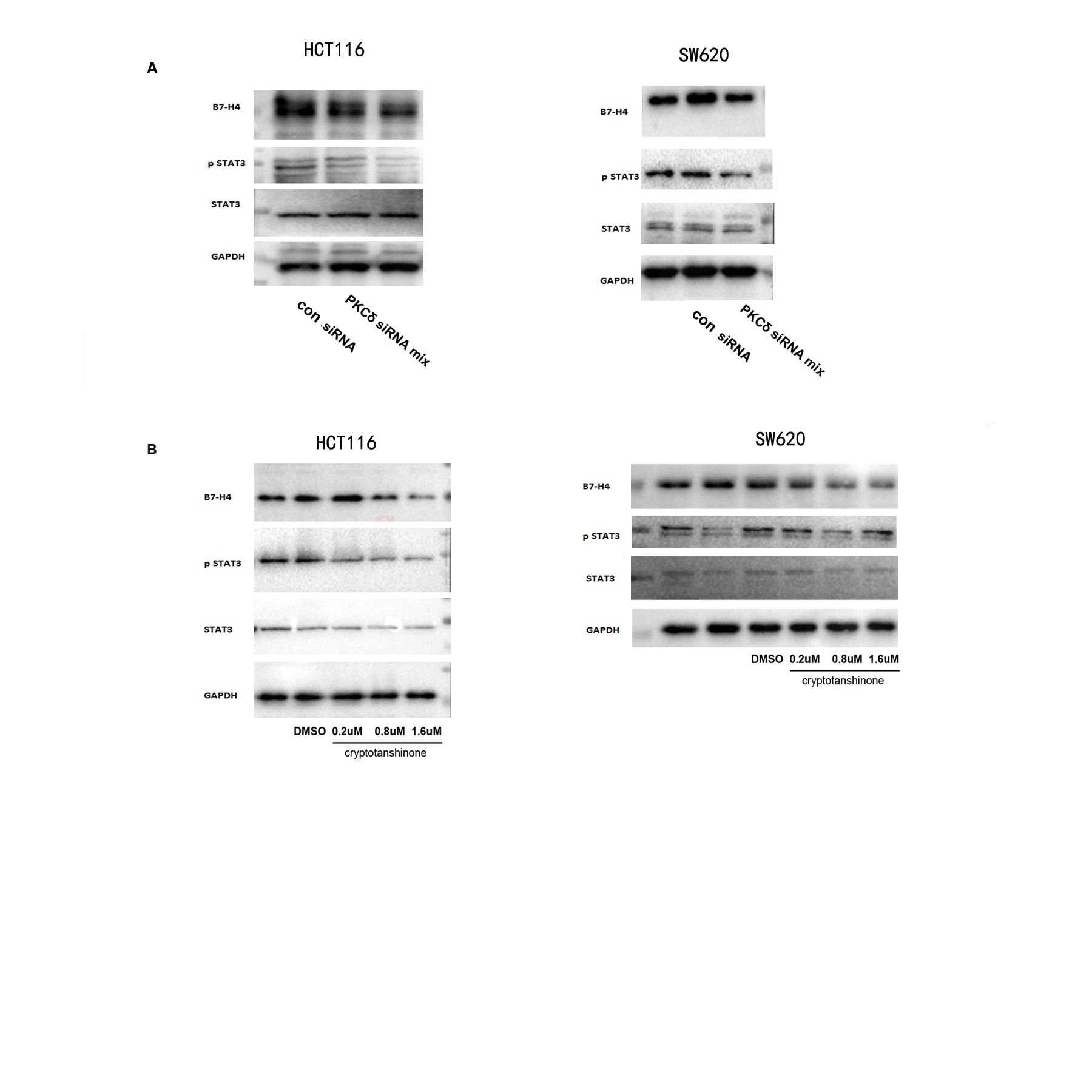

Supplement: Supplementary file 5 — Additional file 5: Figure S5. PKCδ inhibited B7-H4 expression via STAT3 in CRC cell lines. Treatment with a PKCδ-specific siRNA reduced the expression of both B7-H4 and STAT3 in HCT116 and SW620 cells (A). HCT116 and SW620 cells were treated with various concentrations of the STAT3 inhibitor cryptotanshinone (B) for 24 h. The cells were harvested to generate whole-cell lysates for detection of the indicated proteins by Western blot analysis. [file 12935_2022_2567_MOESM5_ESM.tif]

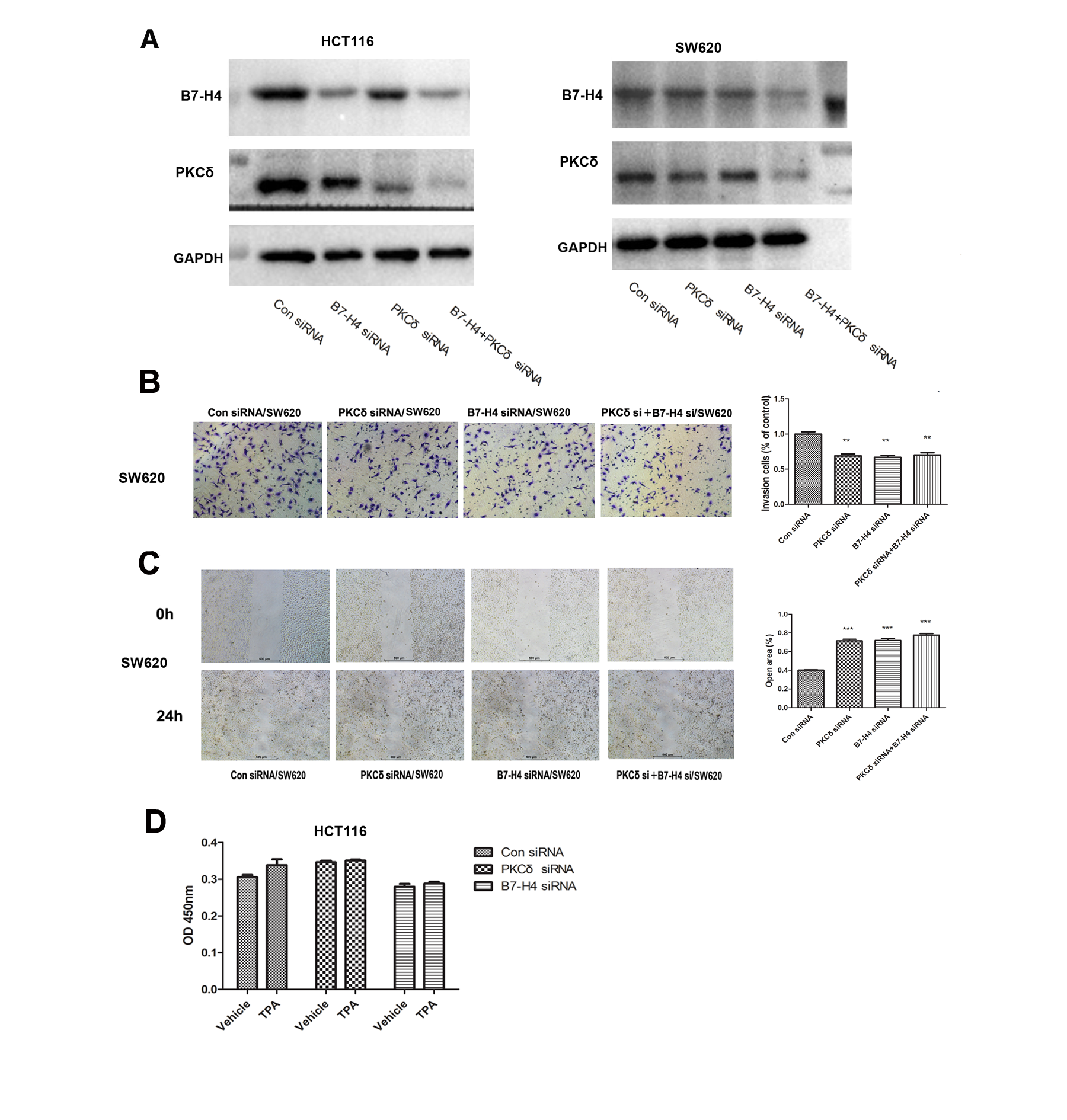

Supplement: Supplementary file 6 — Additional file 6: Figure S6. The PKCδ/B7-H4 axis promoted the migration of SW620 cells. HCT116 and SW620 cells were treated with a PKCδ-specific siRNA and/or a B7-H4-specific siRNA for 45 h, and B7-H4 protein levels were then determined by Western blot analysis (A). A Transwell assay was performed to examine the constitutive invasion of B7-H4 siRNA/SW620, PKCδ siRNA/SW620, PKCδ siRNA + B7-H4 siRNA/SW620 and con siRNA/SW620 cells (B). A wound healing assay was performed to evaluate the effects of PKCδ and B7-H4 on cell migration (C). The viability of HCT116 cells in different groups was assessed by a CCK-8 assay (D). Experiments were performed in triplicate. *P < 0.05, **P < 0.01 and ***P < 0.001. [file 12935_2022_2567_MOESM6_ESM.tif]
