# Supplementary material for: B7-H4 expression is upregulated by PKCδ activation and contributes to PKCδ-induced cell motility in colorectal cancer
Source: Cancer Cell Int. 2022 Apr 11;22:147. doi: 10.1186/s12935-022-02567-1 (PMC8996430; doi:10.1186/s12935-022-02567-1)
Supplement: Supplementary file 7 — Additional file 7: Table S1. The primers of real-time PCR and the siRNAs. [file 12935_2022_2567_MOESM7_ESM.docx]

**Supplemental Table 1** The primers of real-time PCR and the siRNAs

| name | Sequence |
| --- | --- |
| B7-H4 Primer | 5`-AATGAGGGAGTGGAGGAG-3`;  5`- CTGGTGCCCGATAGAGTT-3` |
| PKC-δPrimer | 5`-GTGCAGAAGAAGCCGACCAT-3`;  5`-CCCGCATTAGCACAATCTGGA-3` |
| GAPDH Primer  PKC-δ siRNA1  PKC-δ siRNA2  B7-H4 siRNA  con siRNAs | 5`-GTGAAGGTCGGAGTCAACG-3`  5`-TGAGGTCAATGAAGGGGTC-3`  5’-CCCAGAGACUACAGCAACUTT-3’  5’-AGUUGCUGUAGUCUCUGGGTT-3’  5’- AGAAGG AUGUGGUCCUGAUTT-3’  5’-AUCAGGACCACAUCCUUCUTG-3’  5’-CUCCAUCACAGUCACUACUTT-3’  5’-AGUAGUGAC UGUGAUGGAGTT-3’  5’-UUC UCCGAACGUGUCACGUTT-3’  5’-ACGUGACACGUUCGGAGAATT-3’ |
